# M-Sec promotes the accumulation of intracellular HTLV-1 Gag puncta and the incorporation of Env into viral particles

**DOI:** 10.1371/journal.ppat.1012919

**Published:** 2025-01-27

**Authors:** Masateru Hiyoshi, Youssef M. Eltalkhawy, Randa A. Abdelnaser, Akira Ono, Kazuaki Monde, Yosuke Maeda, Reem M. Mahmoud, Naofumi Takahashi, Yasuyoshi Hatayama, Akihide Ryo, Satoshi Nozuma, Hiroshi Takashima, Ryuji Kubota, Shinya Suzu

**Affiliations:** 1 Research Center for Biological Products in the Next Generation, National Institute of Infectious Diseases, Tokyo, Japan; 2 Joint Research Center for Human Retrovirus Infection, Kumamoto University, Kumamoto, Japan; 3 Department of Microbiology and Immunology, University of Michigan Medical School, Ann Arbor, Michigan, United States of America; 4 Department of Microbiology, Faculty of Life Sciences, Kumamoto University, Kumamoto, Japan; 5 Department of Virology III, National Institute of Infectious Diseases, Tokyo, Japan; 6 Department of Neurology and Geriatrics, Kagoshima University Graduate School of Medical and Dental Sciences, Kagoshima, Japan; 7 Joint Research Center for Human Retrovirus Infection, Kagoshima University, Kagoshima, Japan; Imperial College London Faculty of Medicine, UNITED KINGDOM OF GREAT BRITAIN AND NORTHERN IRELAND

## Abstract

We have demonstrated that the cellular protein M-Sec promotes the transmission of human T-cell leukemia virus type 1 (HTLV-1) in vitro and in vivo. Here, we show how HTLV-1 utilizes M-Sec for its efficient transmission. HTLV-1-infected CD4^+^ T cells expressed M-Sec at a higher level than uninfected CD4^+^ T cells. The ex vivo culture of the infected cells upregulated the expression of M-Sec, the level of which was sustained for a long time. The viral structural protein Gag is distributed in a punctate pattern in cells. M-Sec promoted the accumulation of large intracellular Gag puncta. This accumulation was dependent on phosphatidylinositol 4,5-bisphosphate (PIP2), since it was lost upon the removal of PIP2 binding motifs in M-Sec or the depletion of cellular PIP2. The viral envelope protein Env co-localized with the large Gag puncta induced by M-Sec. Furthermore, viral particles produced by M-Sec-expressing cells contained a higher amount of Env. Given that M-Sec alters the cellular distribution of PIP2, these results suggest that M-Sec promotes the formation of infectious viral particles through PIP2. Since the expression of M-Sec is mediated by HTLV-1 Tax protein, M-Sec appears to function in a positive feedback loop that ensures efficient HTLV-1 transmission.

## Author summary

Human T-cell leukemia virus type 1 (HTLV-1) causes an aggressive blood cancer known as ATL and a neurodegenerative condition known as HAM/TSP. HTLV-1 infects CD4^+^ T lymphocytes, and induces or upregulates the expression of a wide array of cellular proteins, including M-Sec. Our previous in vitro and in vivo studies have demonstrated that M-Sec mediates an efficient cell-to-cell transmission of HTLV-1. In this study, we discovered that M-Sec can also facilitate virus spread by promoting the formation of infectious viral particles. Mechanistically, our data suggest that through redistribution of phosphatidylinositol 4,5-bisphosphate, M-Sec promotes accumulation of a viral structural protein Gag and an envelope protein Env to large intracellular compartments, and facilitates the incorporation of Env into progeny viral particles. Thus, the current study reveals that M-Sec and its downstream effectors are key host components that govern both the virion infectivity and cell-to-cell transmission and thereby potentially serve as targets of anti-HTLV-1 strategies.

## Introduction

The infection of human T-cell leukemia virus type 1 (HTLV-1) is asymptomatic in most cases, but the virus causes at least two distinct diseases, an aggressive blood cancer known as adult T-cell leukemia/lymphoma (ATL), and a neurodegenerative condition known as HTLV-1-associated myelopathy/tropical spastic paraparesis (HAM/TSP). HTLV-1 preferentially infects CD4^+^ T cells in vivo, and cell-to-cell infection is recognized as a central route for the transmission of HTLV-1 because the cell-free infection is quite inefficient [1–5]. Thus, it is important to fully understand the process of cell-to-cell infection of HTLV-1.

M-Sec (also known as TNFα-induced protein 2, tnfaip2) is the 74-kDa cytosolic protein with no known enzymatic activity. The well-known function of M-Sec is the formation of tunneling nanotubes TNTs [6,7], which are the F-actin^+^ long plasma membrane protrusions which often connect distant cells [8,9]. M-Sec is also known to enhance cell motility [10,11]. To initiate TNT formation, M-Sec requires RalA and the exocyst complex [6,7]. RalA is a small GTPase, and the exocyst complex is the evolutionary conserved octameric protein complex involved in vesicle trafficking, such as the tethering of secretary vesicles to the plasma membrane [12,13]. The precise mechanisms by which M-Sec initiates TNT formation and enhances cell motility through RalA and the exocyst complex are not fully understood. Meanwhile, these two functions of M-Sec are beneficial for transmission of pathogens, since both TNTs that connect distant cells and the enhanced motility of infected cells may increase the likelihood of encountering target cells and contact between those cells, thereby facilitating cell-to-cell infection.

In fact, we have demonstrated that M-Sec promotes cell-to-cell infection of human immunodeficiency virus type 1 (HIV-1) in macrophages [14,15], and HTLV-1 in CD4^+^ T cells [16]. Under physiological conditions, M-Sec is highly expressed in myeloid cells including monocytes and macrophages, but not in lymphoid cells including CD4^+^ T cells [6]. We initially found that CD4^+^ T cells of asymptomatic carriers of HTLV-1, but not those of HTLV-1^-^ individuals, expressed M-Sec after an ex vivo culture [16]. In several in vitro co-culture assay systems, the knockdown or pharmacological inhibition of M-Sec in HTLV-1^+^ cells reduced viral transfer to target cells [16]. When humanized mice were inoculated with either control or M-Sec knockdown HTLV-1^+^ cells, the number of HTLV-1 proviral copies in human T cells in the liver, spleen, bone marrow, and peripheral blood of the M-Sec knockdown group were much lower than that of the control group [16]. These results suggest that M-Sec mediates efficient cell-to-cell infection of HTLV-1 in vitro and in vivo. However, it is unclear whether the formation of TNTs and enhanced cell motility fully explain the function of M-Sec.

Of importance, the M-Sec knockdown or pharmacological inhibition not only reduced TNT formation and cell motility, but also altered the intracellular distribution of the viral protein Gag [16]. Gag is the important structural protein, and its assembly is the initial step requisite for the formation of viral particles [1,17–21]. In HTLV-1^+^ T cells, Gag forms many puncta which often accumulate to the perinuclear region and form large puncta [16]. M-Sec knockdown/inhibition reduced the number of the large Gag puncta [16]. Thus, it is likely that M-Sec mediates efficient cell-to-cell infection of HTLV-1 through multiple mechanisms. Among them, the accumulation of Gag puncta may be most important, since it may directly relate to viral particle production. However, how M-Sec promotes the accumulation of Gag puncta and whether it also affects subsequent steps toward viral particle formation are unknown.

Here, we report that HTLV-1 infection causes a sustained expression of M-Sec in CD4^+^ T cells, and that M-Sec promotes the accumulation of Gag puncta through a phosphoinositide and thereby the formation of infectious viral particles.

## Results

### The induction of M-Sec by Tax is mechanistically different from that of 4-1BB or HIAP1

We initially examined how HTLV-1 induces M-Sec expression in T cells. Tax is the transcriptional transactivator protein of HTLV-1, and plays a central role in the viral gene expression. When expressed in the T cell line Jurkat (S1A Fig), Tax induced M-Sec and other well-known Tax-inducible genes, 4-1BB (also known as CD137 or TNFRSF9) and HIAP1 (also known as cIAP2 or BIRC3) [22,23]. The M7 and M22 mutants, both of which are defective in the activation of NF-κB [24], did not induce any of the three genes (S1A Fig). In contrast, the M47 mutant, which can activate NF-κB but not MAP kinases [24], induced 4-1BB and HIAP1 strongly, but M-Sec modestly (S1A Fig). The expression level of the M47 mutant was comparable to that of the wild-type (S1B Fig). Thus, these results implies that the induction of M-Sec by Tax is mechanistically different from that of 4-1BB or HIAP1.

### HTLV-1 infection causes a sustained expression of M-Sec in CD4^+^ T cells

**Fig 1:**
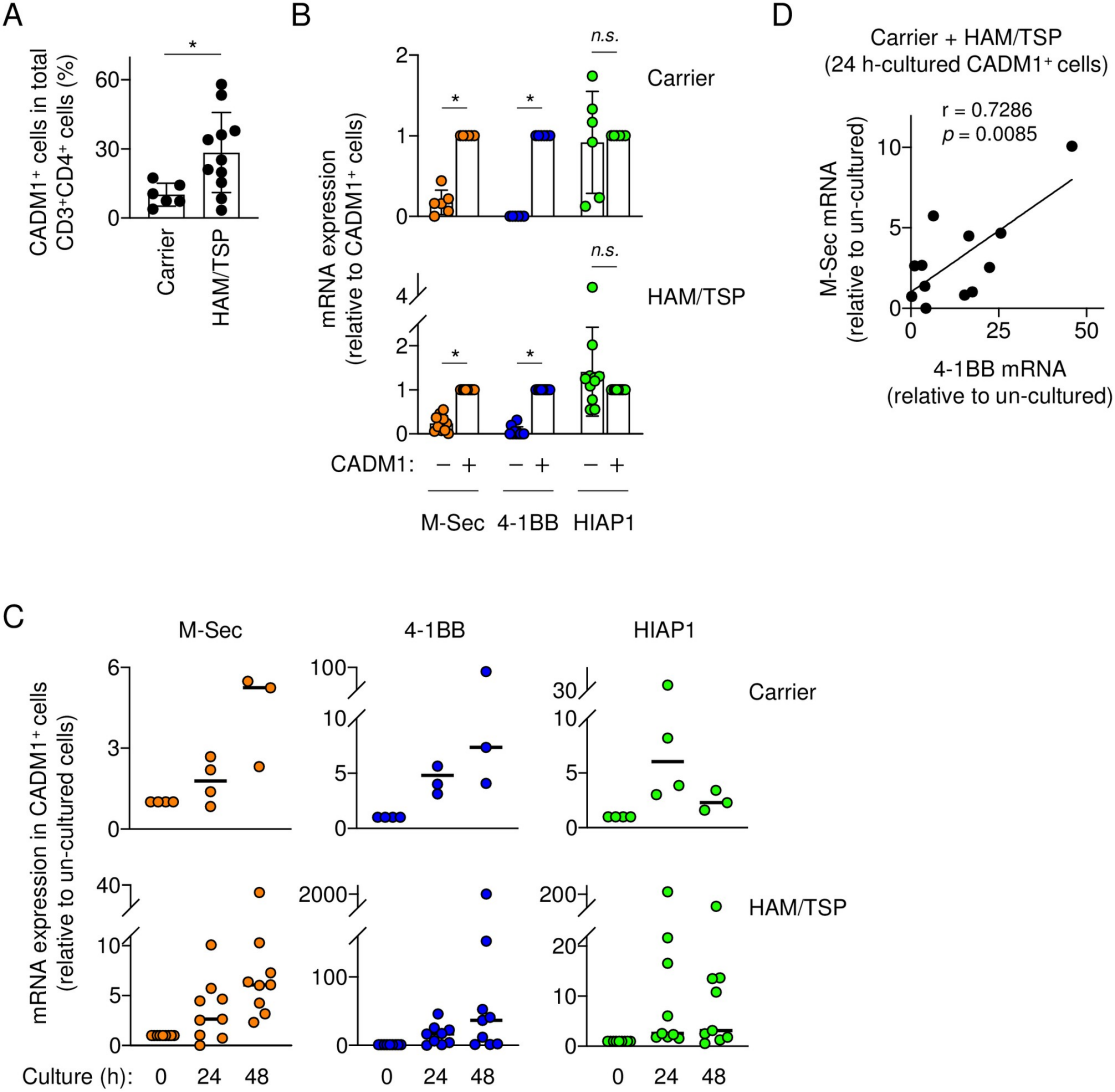
Expression of M-Sec, 4-1BB, or HIAP1 in freshly-isolated or ex vivo cultured CD4^+^ T cells of HTLV-1 carriers and individuals with HAM/TSP. (**A**) Asymptomatic HTLV-1 carriers (n = 6) or individuals with HAM/TSP (n = 11) were analyzed for the percentage of CADM1^+^ cells in the total CD3^+^CD4^+^ cells by flow cytometry. (**B**) The CD3^+^CD4^+^CADM1^+^ cells or CD3^+^CD4^+^CADM1^-^ cells sorted from PBMCs of carriers (n = 6, upper) or individuals with HAM/TSP (n = 11, lower) were analyzed for the mRNA expression of M-Sec, 4-1BB, or HIAP1 by qRT-PCR. The expression level shown is relative to that of CD3^+^CD4^+^CADM1^+^ cells. (**C**) The CD3^+^CD4^+^CADM1^+^ cells were sorted from PBMCs of carriers (n = 3 or 4) or individuals with HAM/TSP (n = 9), and left un-cultured or cultured for 24 or 48 h. The cells were analyzed for the mRNA expression of M-Sec, 4-1BB, or HIAP1 by qRT-PCR. The expression level shown is relative to that of un-cultured cells. (**D**) The CD3^+^CD4^+^CADM1^+^ cells sorted from PBMCs of HTLV-1^+^ individuals (n = 12; three carriers and nine individuals with HAM/TSP) were left un-cultured or cultured for 24 h, and analyzed for the mRNA expression of M-Sec or 4-1BB by qRT-PCR. The fold-induction values of M-Sec and 4-1BB mRNAs were subjected to Pearson correlation analysis. *n*.*s*., not significant. *p < 0.05.

We next examined whether the expression of M-Sec, 4-1BB, and HIAP1 is also differently regulated in HTLV-1-infected primary cells. To this end, peripheral blood mononuclear cells (PBMCs) of asymptomatic carriers and individuals with HAM/TSP were collected, and the CD3^+^CD4^+^ cells expressing CADM1 (also known as TSLC1) were analyzed as HTLV-1-infected cells [25]. The number of CD3^+^CD4^+^CADM1^+^ cells of individuals with HAM/TSP was higher than that of carriers (Fig 1A). In both groups, the freshly-isolated CADM1^+^ T cells expressed M-Sec and 4-1BB at higher levels than the freshly-isolated CADM1^-^ T cells (Fig 1B). Such difference was not observed for HIAP1 (Fig 1B). Because Tax is expressed in intermittent bursts [26,27], Tax-expressing cells are rarely detected in freshly-isolated CD4^+^ T cells and become detectable after the ex vivo culture. In fact, the ex vivo culture upregulated the expression of M-Sec, 4-1BB, and HIAP1 in CADM1^+^ T cells (Fig 1C). Among the three genes, 4-1BB was upregulated most strongly, albeit variably in HTLV-1^+^ individuals (Fig 1C). Despite the variability, the M-Sec induction tended to correlate the 4-1BB induction (Fig 1D).

**Fig 2:**
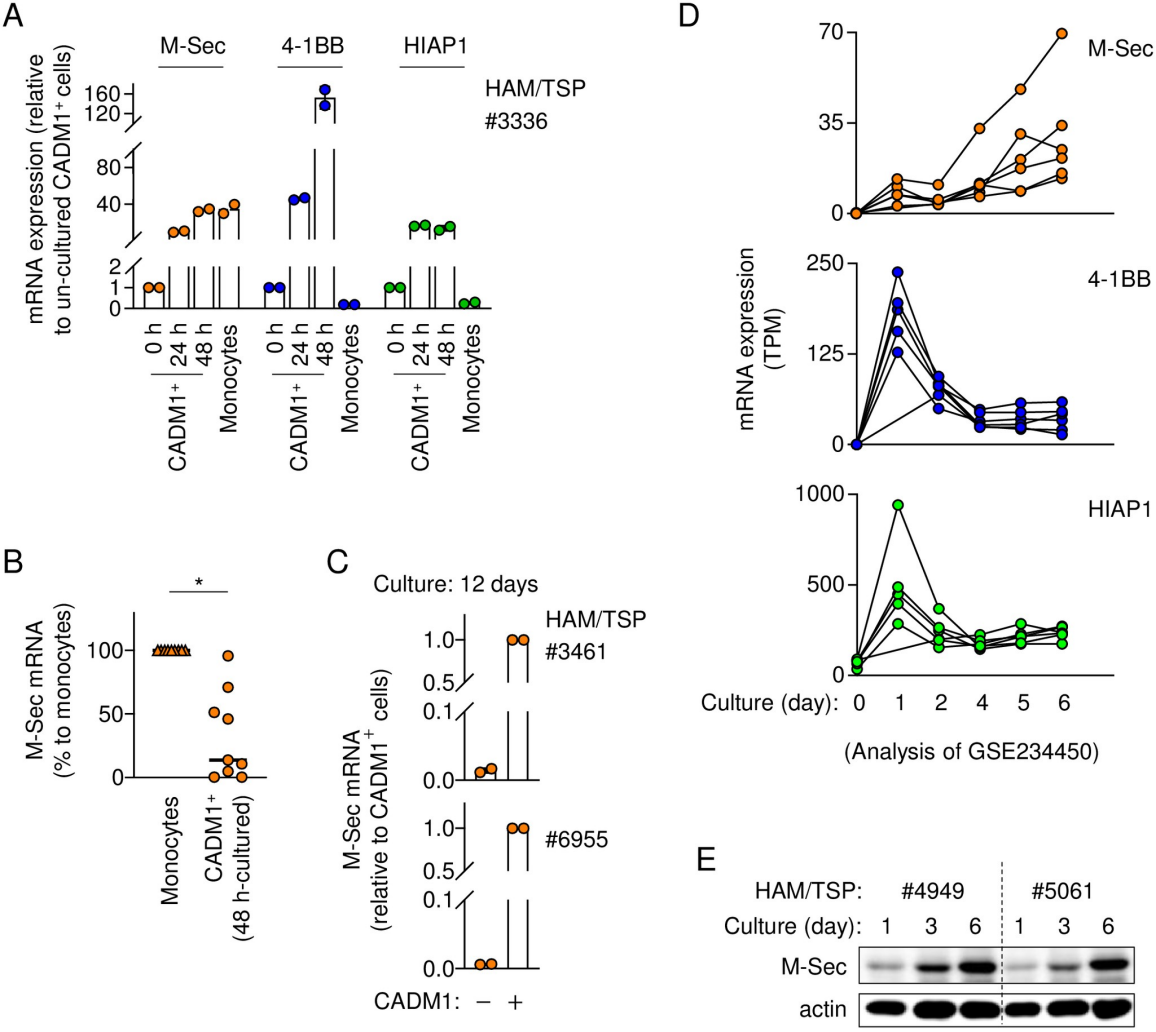
Sustained expression of M-Sec in cultured CD4^+^ T cells of HTLV-1^+^ individuals. (**A**) The CD3^+^CD4^+^CADM1^+^ cells sorted from PBMCs of an individual with HAM/TSP (#3336) were left un-cultured or cultured for 24 or 48 h, and analyzed for the mRNA expression of M-Sec, 4-1BB, or HIAP1 by qRT-PCR. Monocytes of the same individual were also analyzed as a control. The expression level shown is relative to that of un-cultured CD3^+^CD4^+^CADM1^+^ cells. (**B**) The CD3^+^CD4^+^CADM1^+^ cells sorted from PBMCs of individuals with HAM/TSP (n = 9) were cultured for 48 h and analyzed for M-Sec mRNA expression by qRT-PCR. Monocytes of the same individual were also analyzed as a control. The expression level shown is the percentage to that of monocytes. (**C**) The CD3^+^CD4^+^CADM1^+^ cells sorted from PBMCs of individuals with HAM/TSP (#3461 and #6955) were cultured for 12 days and analyzed for M-Sec mRNA expression by qRT-PCR. The expression level shown is relative to that of un-cultured CD3^+^CD4^+^CADM1^+^ cells. (**D**) The time-course RNA-Seq data, in which Aristodemou et al. cultured CD4^+^ T cells of two HTLV-1 asymptomatic carriers and four individuals with HAM/TSP for 6 days [28], were downloaded (the NCBI Gene Expression Omnibus, #GSE234450) and analyzed for the mRNA expression of M-Sec, 4-1BB, or HIAP1. TPM, transcripts per million. *p < 0.05. (**E**) Monocytes present in PBMCs of individuals with HAM/TSP (#4949 and #5061) were depleted by the adherence to culture dishes for 30 min. The monocytes-depleted PBMCs were cultured with RPMI1640 medium/10% FCS containing rhIL-2 for 1, 3 or 6 days, and analyzed for the expression of M-Sec protein by western blotting. β-actin blot is the loading control.

M-Sec is highly expressed in monocytes but rarely detected in CD4^+^ T cells [6]. However, in several, but not all, individuals with HAM/TSP, the expression level of M-Sec mRNA in the cultured CADM1^+^ T cells was not much different than that in monocytes (Fig 2A and 2B). The high expression level of M-Sec mRNA in CADM1^+^ T cells was observed even after 12 days of the ex vivo culture (Fig 2C). Recently, Aristodemou et al. cultured T cells of two carriers and four individuals with HAM/TSP, and performed a time-course RNA-Seq analysis [28]. When their data deposited in the public NCBI Gene Expression Omnibus (#GSE234450) were analyzed, 4-1BB and HIAP were upregulated as early as 1 day after the ex vivo culture but declined thereafter (Fig 2D), the kinetics of which was similar to that of the expression of Tax mRNA [28]. In contrast, M-Sec mRNA was slowly but steadily upregulated at least up to day 6 (Fig 2D). Consistent with this, M-Sec protein was steadily upregulated at least up to day 6 (Fig 2E). These results suggest that HTLV-1 infection causes a sustained expression of M-Sec in CD4^+^ T cells.

### M-Sec promotes the accumulation of large Gag puncta in both primary CD4^+^ T cells and transfected 293 cells

We previously reported that the formation of intracellular Gag puncta and their accumulation to the perinuclear region in CD3^+^CD4^+^CADM1^+^ cells of HTLV-1 carriers during the ex vivo culture were reduced in the presence of an M-Sec inhibitor [16], which inhibits M-Sec-mediated TNT formation [14,29,30]. The similar result was obtained when CD3^+^CD4^+^CADM1^+^ cells of individuals of HAM/TSP were used: the M-Sec inhibitor reduced the number and size of accumulated Gag puncta, and induced the diffuse Gag signal throughout cells (S2 Fig).

**Fig 3:**
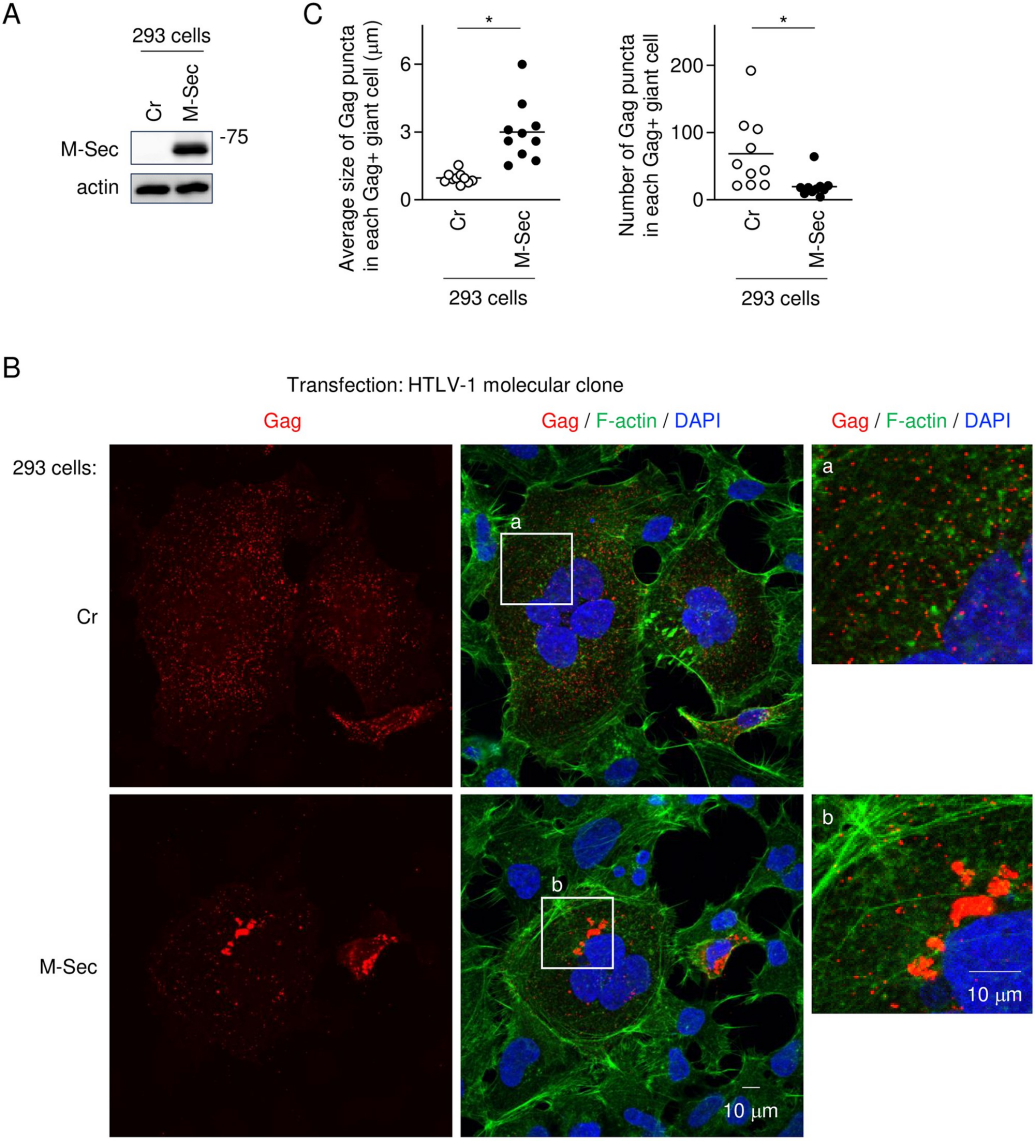
Effect of M-Sec on Gag puncta in 293 cells transfected with HTLV-1 molecular clone. (**A**) The control (Cr) 293 cells or 293 cells stably expressing M-Sec were analyzed for the expression of M-Sec by western blotting. β-actin blot is the loading control. (**B**) Cr-293 cells or M-Sec-293 cells were transfected with the HTLV-1 molecular clone pX1MT-M (1 μg), cultured for 2 days, and analyzed for Gag (red) by immunofluorescence. In middle panels, the nuclei and F-actin were stained with DAPI (blue) and phalloidin (green), respectively. In right panels, the magnified images of "a" and "b" in the middle panels are shown. Scale bar: 10 μm. *p < 0.05. (**C**) The cells were analyzed as in **B**. *Left panel*, the average size of Gag puncta in each Gag^+^ giant cell is summarized (10 cells for each group). *Right panel*, the number of Gag puncta in each Gag^+^ giant cell is summarized (10 cells for each group). The Gag signal larger than approximately 0.5 μm was considered puncta. *p < 0.05.

**Fig 4:**
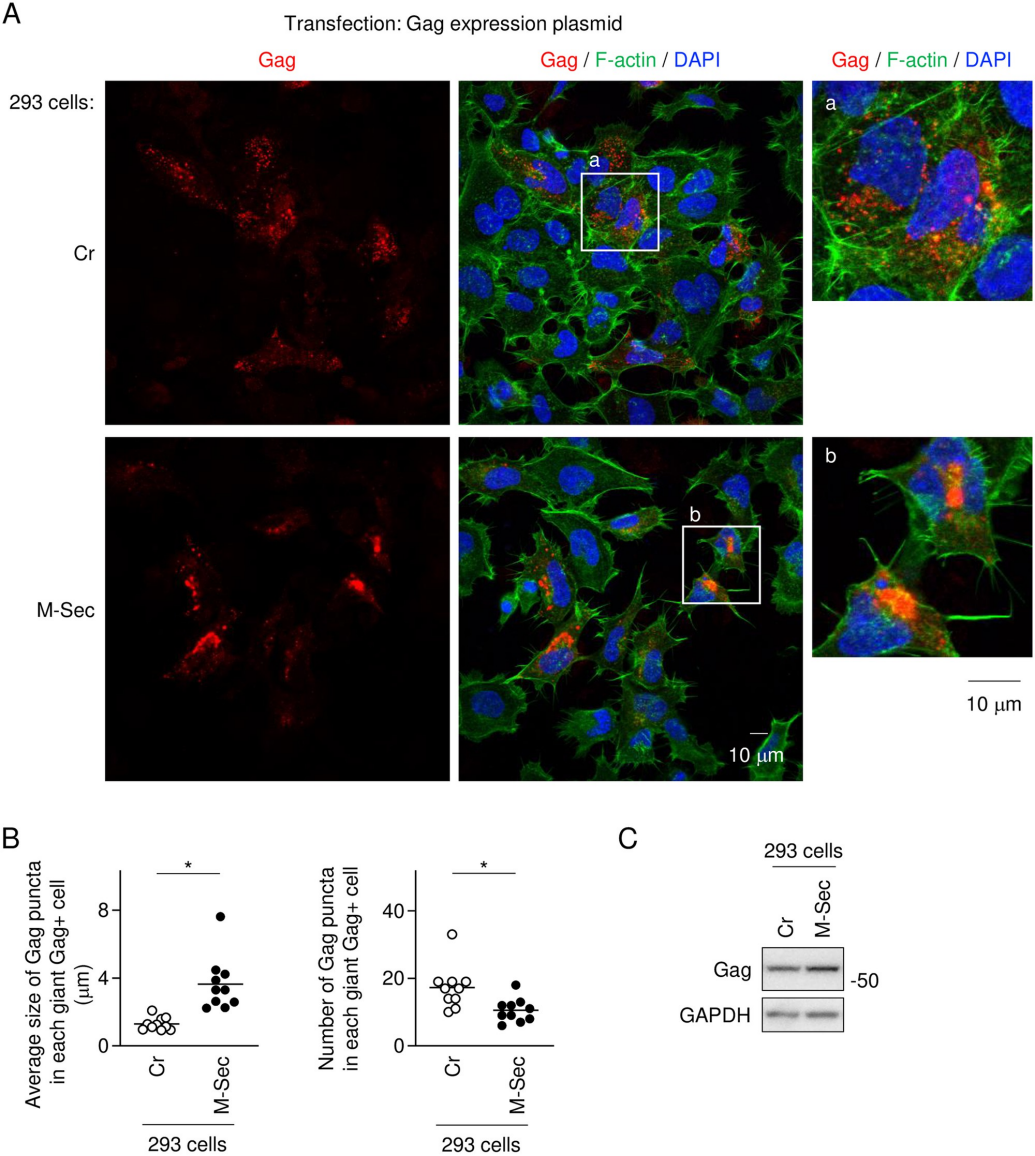
Effect of M-Sec on Gag puncta in 293 cells transfected with Gag expression plasmid. (**A**) The control (Cr) 293 cells or 293 cells stably expressing M-Sec were transfected with the Gag expression plasmid pCRVI/HTLV-1/Gag-Flag (0.3 μg), cultured for 2 days, and analyzed for Gag (red) by immunofluorescence. In middle panels, the nuclei and F-actin were stained with DAPI (blue) and phalloidin (green), respectively. In right panels, the magnified images of "a" and "b" in the middle panels are shown. Scale bar: 10 μm. (**B**) The cells were analyzed as in **A**. *Left panel*, the average size of Gag puncta in each Gag^+^ cell is summarized (10 cells for each group). *Right panel*, the number of Gag puncta in each Gag^+^ cell is summarized (10 cells for each group). The Gag signal larger than approximately 0.5 μm was considered puncta. *p < 0.05. (**C**) Cr-293 cells and M-Sec-293 cells were transfected and cultured as in **A**, and analyzed for the expression of Gag by western blotting. GAPDH blot is the loading control.

To confirm that M-Sec promotes the accumulation of large Gag puncta, we established 293 cells stably expressing M-Sec (Fig 3A). M-Sec-293 cells formed longer membrane protrusions and showed smaller cell surface area than the control (Cr) 293 cells (S3 Fig), which was consistent with the previous findings that the knockdown of M-Sec in the T cell line MT-2 reduced the membrane protrusions and increased the cell surface area [16]. When transfected with the HTLV-1 molecular clone pX1MT-M [31], both Cr-293 and M-Sec-293 cells formed multinucleated giant cells (Fig 3B, middle panels) because HTLV-1 envelope (Env) protein is highly fusogenic [32–34]. However, these cells showed different signals of Gag (Fig 3B, left and right panels). When quantified, the average size of cytoplasmic Gag puncta in each giant M-Sec-293 cell was larger than that in each giant Cr-293 cell (Fig 3C, left), which was associated with a decrease of the total number of Gag puncta in each giant M-Sec-293 cell (Fig 3C, right). Moreover, when transfected with the Gag expression plasmid [35] in place of the molecular clone, M-Sec-293 cells formed larger Gag puncta than Cr-293 cells (Fig 4A). The average size of Gag puncta in each M-Sec-293 cell was larger than that in each Cr-293 cell (Fig 4B, left), which was associated with the decrease of the total number of Gag puncta in each M-Sec-293 cell (Fig 4B, right). The expression level of Gag in M-Sec-293 cells was comparable to that in Cr-293 cells (Fig 4C). These results suggest that M-Sec promotes the accumulation of the large Gag puncta without affecting the expression level of Gag, and that other viral components are dispensable for the function of M-Sec.

### Lysine-rich motif of M-Sec is indispensable for the accumulation of the large Gag puncta

**Fig 5:**
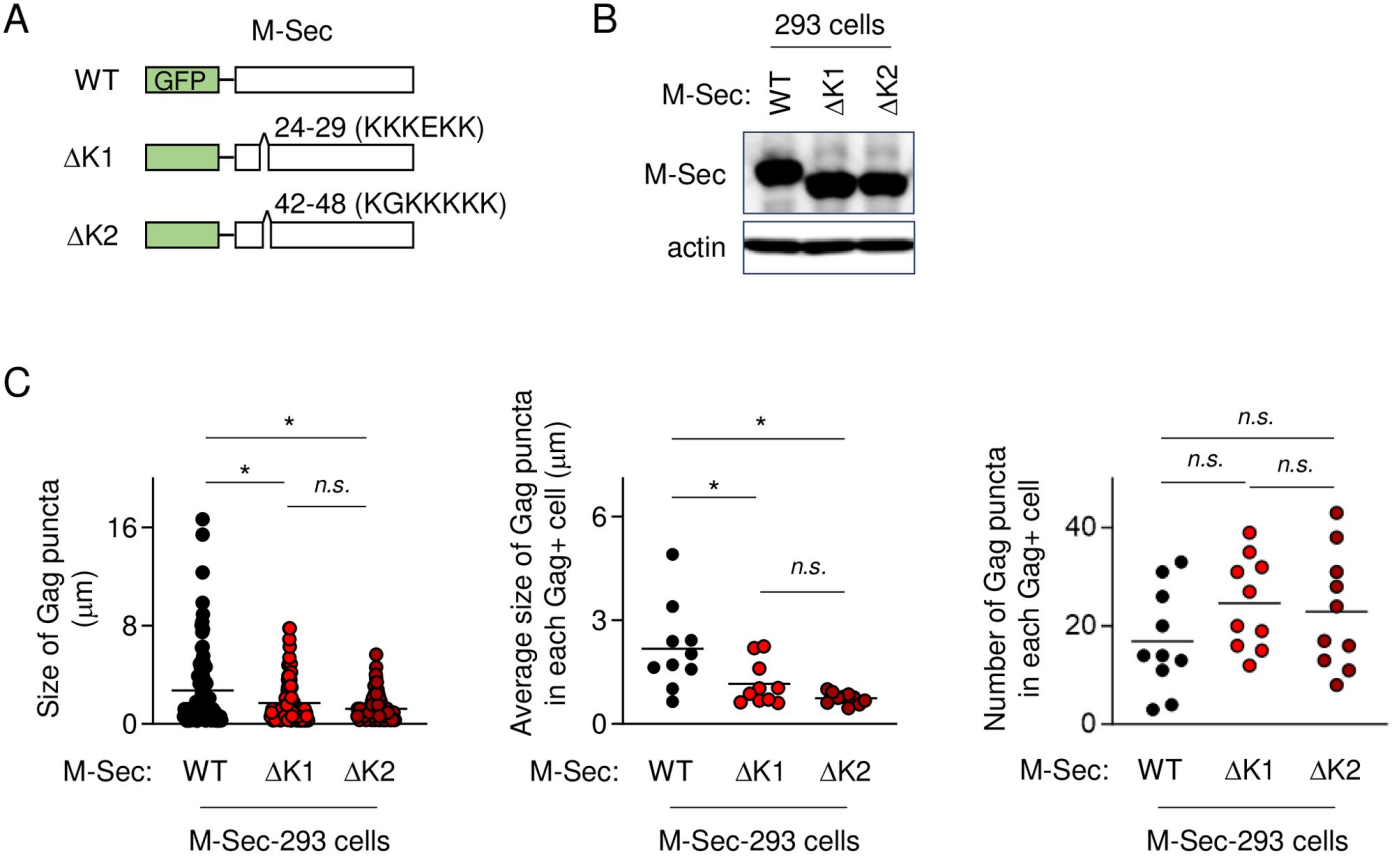
Effect of M-Sec and its mutants on Gag puncta in 293 cells. (**A**) The GFP-fused wild-type (WT) M-Sec and its mutants (ΔK1 and ΔK2) are schematically shown. The ΔK1 and ΔK2 lack the first and second lysine-rich motif, respectively. (**B**) 293 cells were engineered to stably express M-Sec WT, ΔK1, or ΔK2. The expression of these GFP-fused M-Sec proteins was confirmed by western blotting. β-actin blot is the loading control. (**C**) 293 cells, which stably expressed M-Sec WT, ΔK1, or ΔK2 mutant, were transfected with the Gag expression plasmid pCRVI/HTLV-1/Gag-Flag (0.3 μg), cultured for 2 days, and analyzed for Gag by immunofluorescence. *Left panel*, the size of Gag puncta is summarized (100 puncta in randomly selected Gag^+^ cells for each group). *Middle panel*, the average size of Gag puncta in each Gag^+^ cell is summarized (10 cells for each group). *Right panel*, the number of Gag puncta in each Gag^+^ cell is summarized (10 cells for each group). The Gag signal longer than approximately 0.1 μm was considered puncta. *n*.*s*., not significant. *p < 0.05.

We next attempted to clarify mechanism by which M-Sec promotes the accumulation of the large Gag puncta. M-Sec has the polybasic region at its N-terminus that contains two lysine-rich motifs (Fig 5A), and the M-Sec mutant lacking the first or second lysine-rich motif (ΔK1 or ΔK2, see Fig 5A) failed to initiate TNT formation [7]. Thus, we established 293 cells stably expressing the ΔK1 or ΔK2 mutant (Fig 5B). When transfected with the Gag expression plasmid, the size of Gag puncta in cells expressing the ΔK1 or ΔK2 mutant was smaller than that in cells expressing the wild-type (Fig 5C, left). The average size of Gag puncta in each Gag^+^ cell was also small in cells expressing the ΔK1 or ΔK2 mutant (Fig 5C, middle). Instead, the number of Gag puncta in each Gag^+^ cell tended to be high in cells expressing the ΔK1 or ΔK2 mutant, although the difference was not statistically significant (Fig 5C, right). These results indicate that the lysine-rich motif of M-Sec is indispensable for the accumulation of the large Gag puncta.

### M-Sec promotes the accumulation of the large Gag puncta in a manner dependent on phosphatidylinositol 4,5-bisphosphate

**Fig 6:**
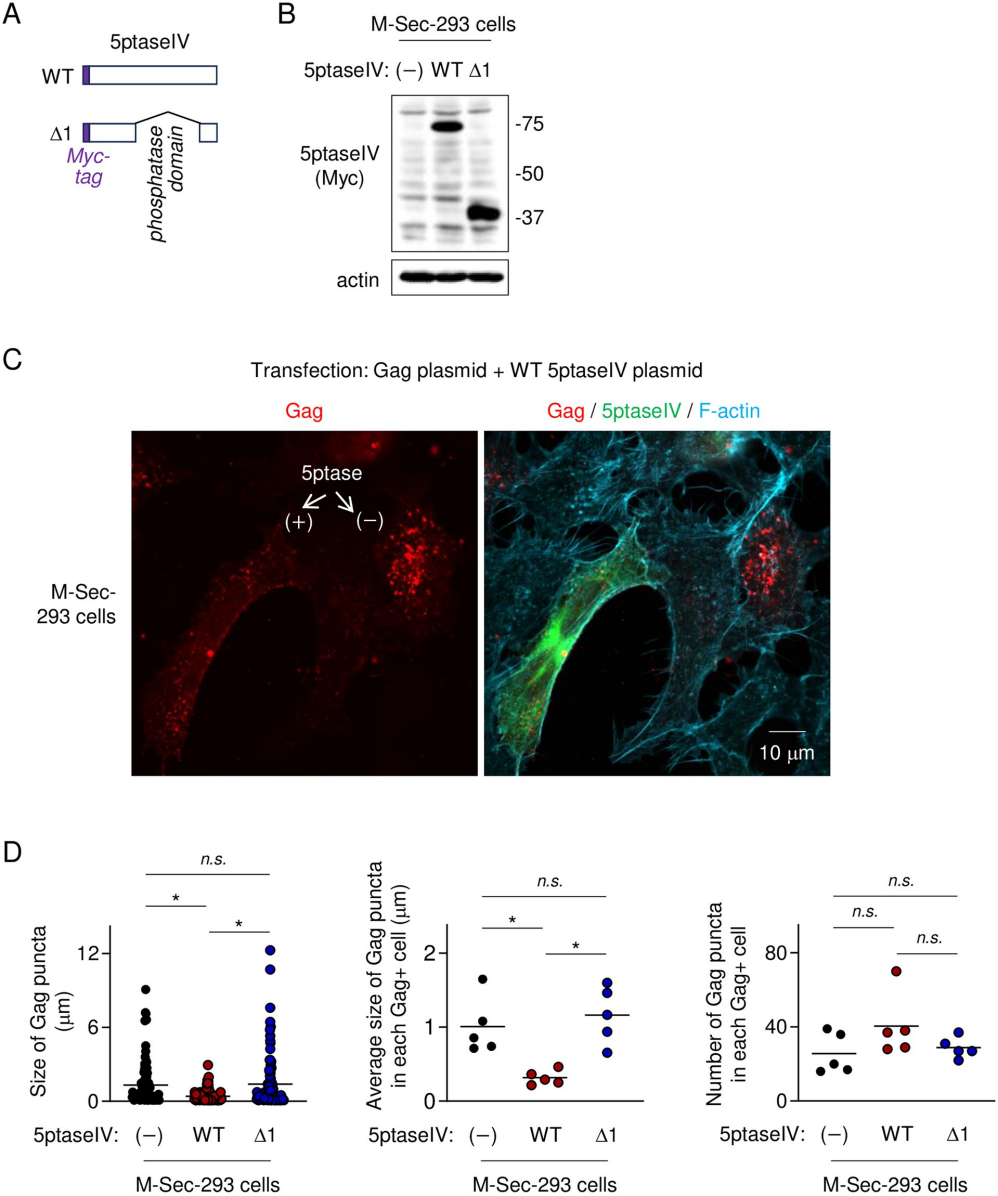
Effect of 5ptaseIV on M-Sec-mediated accumulation of the large Gag puncta in 293 cells. (**A**) The Myc-tagged wild-type (WT) 5ptaseIV and its mutant (Δ1) are schematically shown. The Δ1 lacks the 5-phosphatase domain. (**B**) The 293 cells stably expressing M-Sec (M-Sec-293) were transfected with the empty vector (-), WT or Δ1 5ptaseIV plasmid (0.5 μg), cultured for 2 days, and analyzed for the expression level of these Myc-tagged 5ptaseIV proteins by western blotting. β-actin blot is the loading control. (**C**) M-Sec-293 cells were co-transfected with the Gag expression plasmid pCRVI/HTLV-1/Gag-HA (0.5 μg) and the WT 5ptaseIV plasmid (0.5 μg), cultured for 2 days, and analyzed for Gag (red), 5ptaseIV (green) or F-actin (blue) by immunofluorescence. The 5ptaseIV-positive (+) and negative (−) cells are indicated by arrows. (**D**) M-Sec-293 cells were co-transfected with the Gag expression plasmid pCRVI/HTLV-1/Gag-Flag (0.5 μg), and the empty vector, WT or Δ1 5ptaseIV plasmid (0.5 μg), cultured for 2 days, and analyzed for Gag by immunofluorescence. *Left panel*, the size of Gag puncta is summarized (100 puncta in randomly selected Gag^+^ cells for each group). *Middle panel*, the average size of Gag puncta in each Gag^+^ cell is summarized (5 cells for each group). *Right panel*, the number of Gag puncta in each Gag^+^ cell (5 cells for each group) is summarized. The Gag signal longer than approximately 0.1 μm was considered puncta. *n*.*s*., not significant. *p < 0.05.

It was reported that the lysine-rich motifs of M-Sec binds phosphatidylinositol 4,5-bisphosphate (PIP2) [7], which is the most abundant phosphoinositide and concentrated usually in the plasma membrane [36–38]. Thus, we next examined whether PIP2 is involved in the M-Sec-mediated accumulation of the large Gag puncta. To this end, we used the plasmid expressing inositol polyphosphate 5-phosphatase type IV (5ptaseIV), which reduces cellular level of PIP2 by cleaving the phosphate group at the D5 position of PIP2 [39,40]. As a reference, we used the mutant lacking the 5-phosphatase domain (Δ1) (Fig 6A and 6B). When M-Sec-293 cells were co-transfected with the Gag expression plasmid and the wild-type 5ptaseIV plasmid, the size of Gag puncta became smaller (Fig 6C and 6D, left). The average size of Gag puncta in each Gag^+^ cell also became small (Fig 6D, middle). Instead, the number of Gag puncta in each Gag^+^ cell tended to become high, although the difference was not statistically significant (Fig 6D, right). Such changes were not observed when the Δ1 mutant was used (Fig 6D).

**Fig 7:**
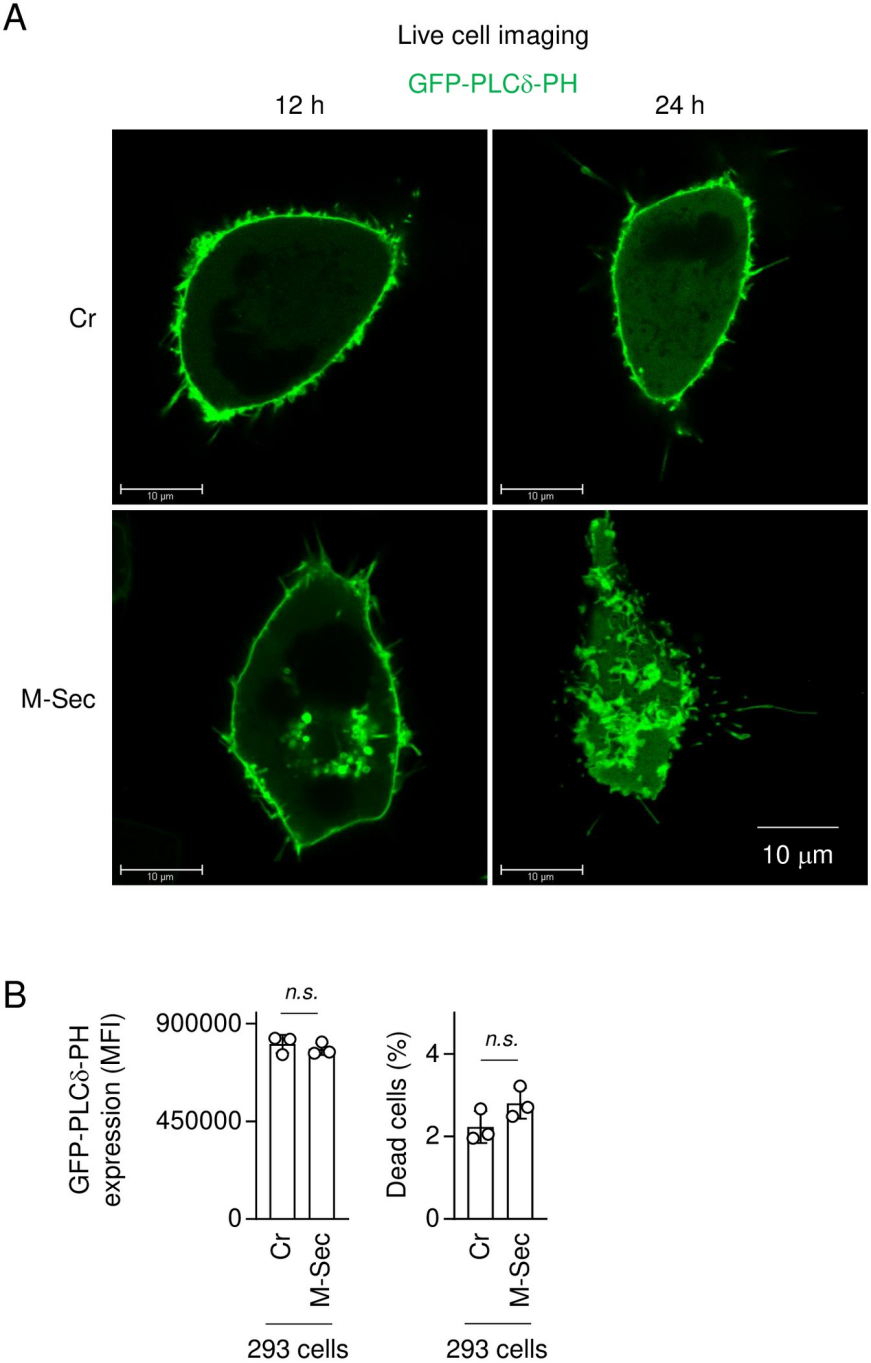
Effect of M-Sec on cellular distribution of PIP2 in 293 cells. (**A**) The control (Cr) 293 cells (upper panels) or 293 cells stably expressing M-Sec (lower panels) were transfected with the GFP-PLCδ-PH plasmid (1 μg), cultured for 12 h (left panels) or 24 h (right panels), and analyzed for GFP expression by the live cell imaging to visualize the cellular distribution of PIP2. Scale bar: 10 μm. (**B**) Cr-293 cells or M-Sec-293 cells were transfected with the GFP-PLCδ-PH plasmid (1 μg), cultured for 24 h, and analyzed for the expression of GFP (to detect GFP-PLCδ-PH) (left panel) or the percentage of dead cells (right panel) by flow cytometry (n = 3). In the left panel, the mean fluorescence intensity (MFI) of GFP is shown. *n*.*s*., not significant.

To further confirm the involvement of PIP2 in the M-Sec-mediated accumulation of large Gag puncta, we used the plasmid expressing GFP-fused pleckstrin homology domain of phospholipase Cδ (GFP-PLCδ-PH), which is widely utilized as a probe to visualize the distribution of PIP2 in living cells [41,42]. When transfected with the GFP-PLCδ-PH plasmid and analyzed by the live cell imaging, Cr-293 cells and M-Sec-293 cells showed a marked difference in the distribution of GFP-PLCδ-PH (Fig 7A): the large GFP-PLCδ-PH-positive structures appeared in M-Sec-293 cells as early as 12 h after the transfection and increased over time (Fig 7A, lower panels). There was no difference in the expression level of GFP-PLCδ-PH and the percentage of dead cells between Cr-293 cells and M-Sec-293 cells (Fig 7B). These results suggest that M-Sec promotes the redistribution of PIP2 itself. Gag of many retroviruses have the polybasic region and bind PIP2 [37], and the binding of HIV-1 Gag with PIP2 regulates its targeting to the plasma membrane [40]. Thus, it appears that M-Sec alters the localization of PIP2 and thereby promotes the accumulation of large cytoplasmic HTLV-1 Gag puncta.

### M-Sec promotes co-localization of Env with cytoplasmic Gag puncta

**Fig 8:**
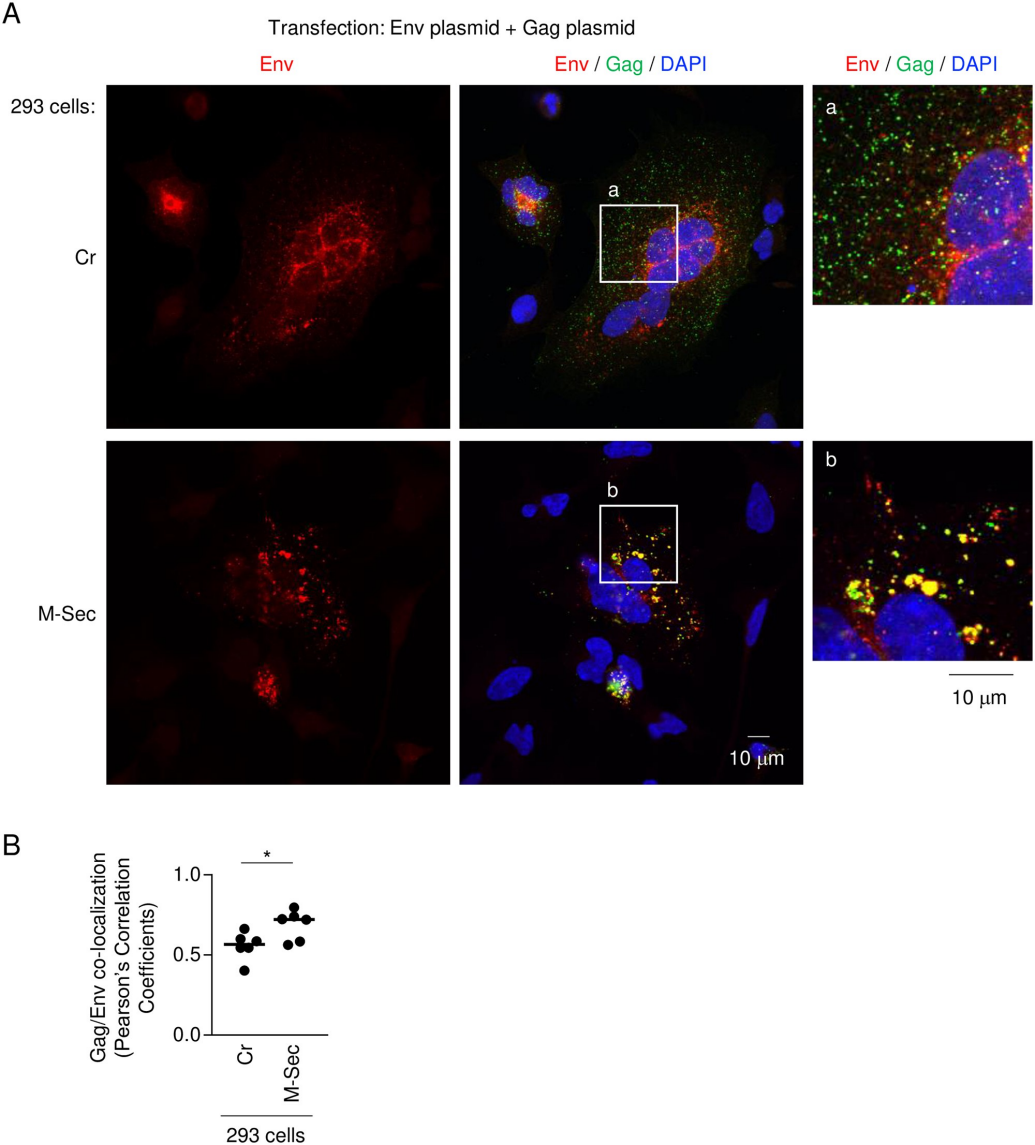
Localization of Env in M-Sec-expressing 293 cells co-transfected with Env- and Gag plasmids. (**A**, **B**) The control (Cr) 293 cells or 293 cells stably expressing M-Sec were co-transfected with the Gag expression plasmid pCRVI/HTLV-1/Gag-Flag (0.3 μg), the Env expression plasmid pcDNA-1E-RRE (0.3 μg) and the Rev expression plasmid pRSV-Rev (0.4 μg), cultured for 2 days, and analyzed for Env (red) or Gag (green) by immunofluorescence. In middle panels, the nuclei were stained with DAPI (blue). In right panels, the magnified images of "a" and "b" in the middle panels are shown. Scale bar: 10 μm. In **B**, Pearson’s correlation coefficients between Gag and Env are shown. *p < 0.05.

We next examined whether the M-Sec-mediated accumulation of the large Gag puncta affects the subsequent steps toward viral particle formation. In particular, we focused on Env because HTLV-1 infects target cells through Env. As explained earlier (see Fig 3), both Cr-293 cells and M-Sec-293 cells formed multinucleated giant cells when transfected with the molecular clone because HTLV-1 Env is highly fusogenic (S4 Fig, middle panels) [32–34]. Of interest, these cells showed different Env signals: there were many but small Env puncta in Cr-293 cells whereas there were a few but large Env puncta in M-Sec-293 cells (S4 Fig, left panels). The large Env-positive compartments in M-Sec-293 cells overlapped with the large Gag puncta (S4 Fig, right panels). However, when transfected with the Env expression plasmid alone, there was no obvious difference in the Env signals between Cr-293 cells and M-Sec-293 cells (S5 Fig). However, once again, when co-transfected with the Env expression plasmid and Gag expression plasmid, there were a few but large Env-positive puncta in M-Sec-293 cells (Fig 8A, left panels), and the large Env puncta overlapped with the large Gag puncta (Fig 8A, right panels and 8B). These results suggest that M-Sec promotes the co-localization of Env with Gag at the large cytoplasmic puncta.

### M-Sec promotes Env incorporation into viral particles

**Fig 9:**
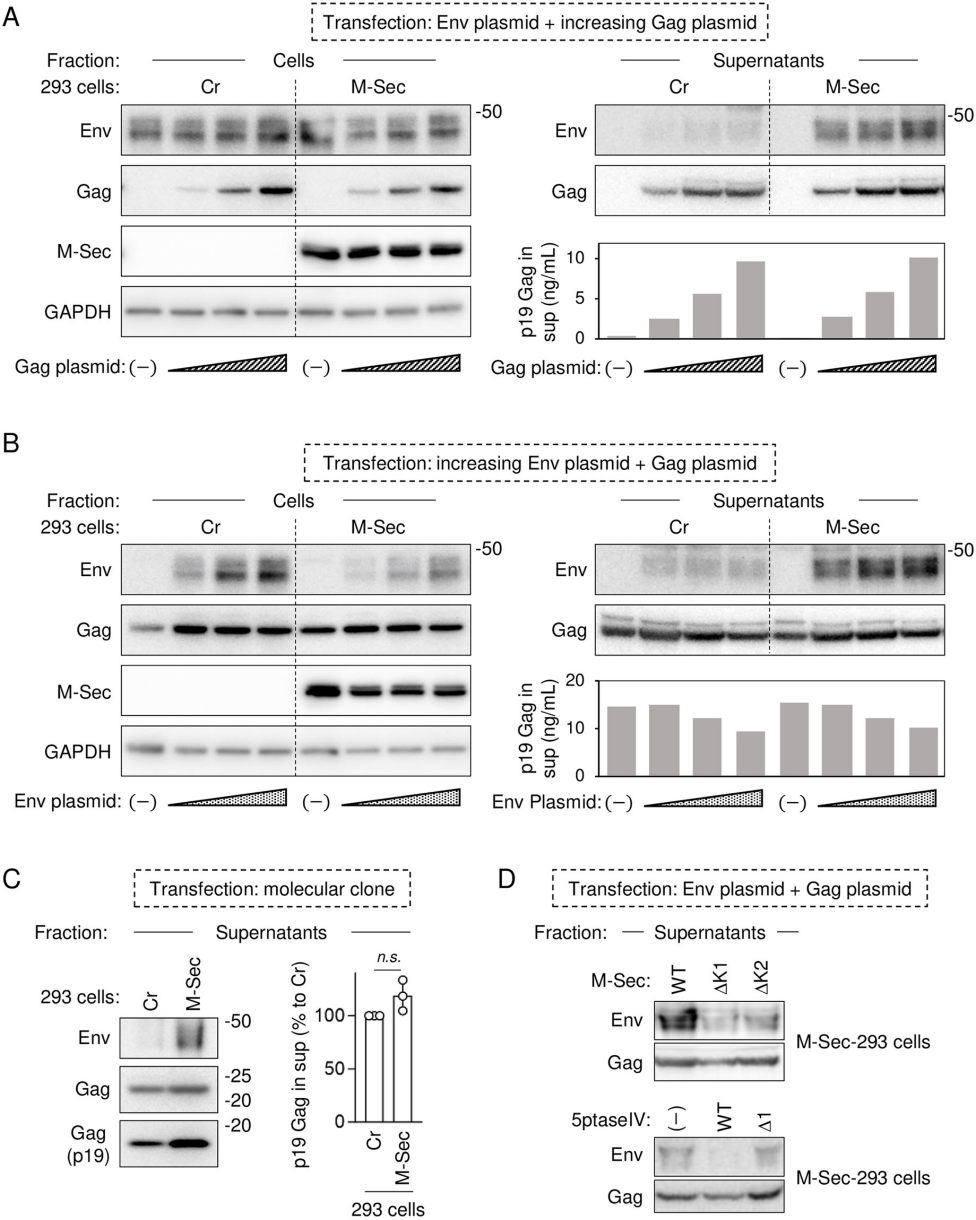
Effect of M-Sec on Env incorporation into virus-like particles produced from 293 cells. (**A**) The control (Cr) 293 cells or 293 cells stably expressing M-Sec were co-transfected with the increasing amount of the Gag expression plasmid (0, 0.1, 0.2, or 0.3 μg), the Env expression plasmid (0.3 μg) and the Rev expression plasmid pRSV-Rev (0.4 μg), and cultured for 2 days. *Left half*, the cells were analyzed for the expression of Env, Gag or M-Sec by western blotting. GAPDH blot is the loading control. *Right half*, the virus-like particles (VLPs) in the supernatants were collected and analyzed for the amount of Env or Gag by western blotting. Also, the supernatants were analyzed for the concentration of p19 Gag by ELISA. (**B**) Cr-293 cells or M-Sec-293 cells were co-transfected with the Gag expression plasmid (0.3 μg), the increasing amount of the Env expression plasmid (0, 0.1, 0.2, or 0.3 μg) and the Rev expression plasmid pRSV-Rev (0.4 μg). Then, the cells were analyzed as in **A**. (**C**) Cr-293 cells or M-Sec-293 cells were transfected with the HTLV-1 molecular clone pX1MT-M (1 μg), and cultured for 2 days. The VLPs in the supernatants were collected and analyzed for the amount of Env or Gag (both p24 and p19) by western blotting. Also, the supernatants were analyzed for the concentration of p19 Gag by ELISA. The level shown is the percentage to that of Cr-293 cells. *n*.*s*., not significant. (**D**) *Upper half*, 293 cells expressing the wild-type (WT) or mutant M-Sec (ΔK1 or ΔK2) were co-transfected with the Gag expression plasmid (0.3 μg), the Env expression plasmid (0.3 μg) and the Rev expression plasmid (0.4 μg), and cultured for 2 days. The VLPs in the supernatants were collected and analyzed for the amount of Env or Gag by western blotting. *Lower half*, 293 cells expressing the wild-type M-Sec were co-transfected with the Gag expression plasmid (0.3 μg), the Env expression plasmid (0.3 μg), the Rev expression plasmid (0.3 μg), and one of the following plasmids; the empty vector (0.1 μg), the wild type 5ptaseIV plasmid (0.1 μg) or the Δ1 mutant 5ptaseIV plasmid (0.1 μg). The cells were cultured for 1 day, and the VLPs in the supernatants were collected and analyzed for the amount of Env or Gag by western blotting.

It is thought that HTLV-1 particles are transmitted in confined areas formed by cell-to-cell contact, and Env incorporation is important for the formation of viral particles. Based on the Gag/Env co-localization (Fig 8 and S4 Fig), we next examined whether M-Sec promotes the Env incorporation into viral particles. First, Cr-293 cells or M-Sec-293 cells were co-transfected with the Env plasmid (0.3 μg) and increasing amount of the Gag plasmid (0, 0.1, 0.2, or 0.3 μg) (Fig 9A, blots in left half). Under the conditions, M-Sec did not affect the viral production because there was no obvious difference in the amount of p19 Gag protein in the supernatants between Cr-293 cells and M-Sec-293 cells (Fig 9A, the graph in right half). In fact, there was no obvious difference in the amount of Gag in the virus-like particles (VLPs) between these cells (Fig 9A, Gag blot in right half). However, the amount of Env in VLPs produced by M-Sec-293 cells was higher than that produced by Cr-293 cells (Fig 9A, Env blot in right half). Such higher amount of Env in VLPs in the presence of M-Sec was confirmed under different conditions, namely, the co-transfection of the Gag plasmid (0.3 μg) and increasing amount of the Env plasmid (0, 0.1, 0.2, or 0.3 μg) (Fig 9B), or the transfection with the HTLV-1 molecular clone (Fig 9C). The amount of Env in VLPs produced by 293 cells expressing the M-Sec mutants (ΔK1 and ΔK2), both of which failed to promote the accumulation of the large Gag puncta (Fig 5), was lower than that produced by 293 cells expressing the wild-type M-Sec (Fig 9D, upper half). The amount of Env in VLPs produced by M-Sec-293 cells transfected with the wild-type 5ptaseIV plasmid, which inhibited the M-Sec-mediated accumulation of large Gag puncta (Fig 6), was also lower than that produced by M-Sec-293 cells transfected with the empty vector or the Δ1 5ptaseIV mutant (Fig 9D, lower half). These results suggest that M-Sec promotes the incorporation of Env into viral particles, which is likely due to the intracellular co-localization of Env with the large Gag puncta induced by M-Sec.

**Fig 10:**
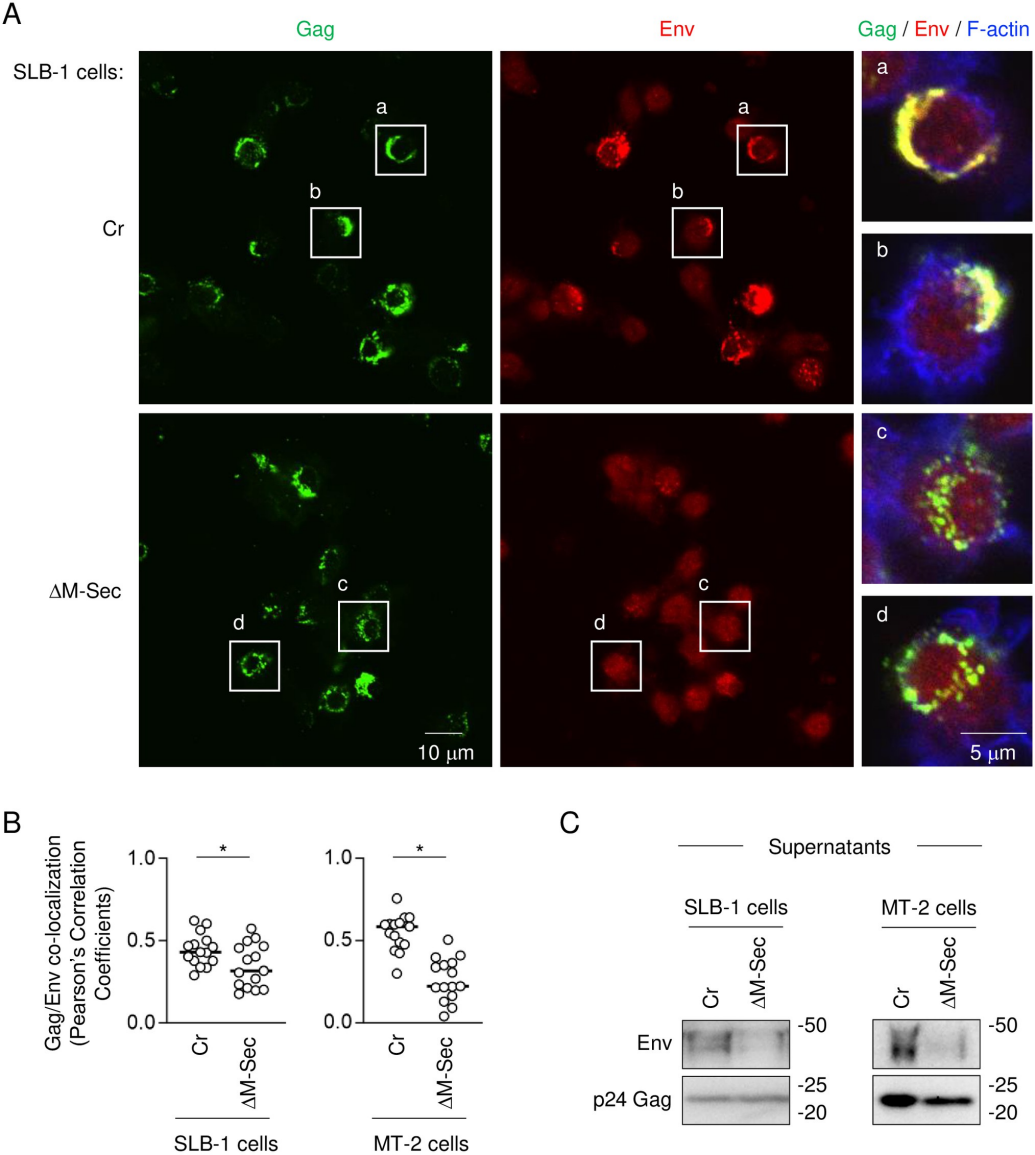
Effect of M-Sec knockdown on Env/Gag co-localization and Env incorporation into virus-like particles in SLB-1 and MT-2 cells. (**A**) The control (Cr) SLB-1 cells or M-Sec knockdown (ΔM-Sec) SLB-1 cells were analyzed for Gag (green), Env (red) or F-actin (blue) by immunofluorescence. In right panels, the magnified images of "a", "b", "c" and "d" in the left and middle panels are shown. Scale bar: 10 μm (left and middle panels) or 5 μm (right panels). (**B**) Pearson’s correlation coefficients between Gag and Env are shown. In the left panel, the control (Cr) SLB-1 cells and M-Sec knockdown (ΔM-Sec) SLB-1 cells were analyzed. In the right panel, the control (Cr) MT-2 cells and M-Sec knockdown (ΔM-Sec) MT-2 cells were analyzed (see S7 Fig for immunofluorescence). *p < 0.05. (**C**) The virus-like particles (VLPs) in the supernatants were collected and analyzed for the amount of Env by western blotting after normalization of the Gag amount. In left blots, the control (Cr) SLB-1 cells and M-Sec knockdown (ΔM-Sec) SLB-1 cells were analyzed. In right blots, the control (Cr) MT-2 cells and M-Sec knockdown (ΔM-Sec) MT-2 cells were analyzed.

Finally, we performed experiments using HTLV-1-infected M-Sec^+^ T cell lines, SLB-1 and MT-2. We previously reported that the knockdown of M-Sec in these cells altered the intracellular localization of Gag [16]. In this study, we found that the M-Sec knockdown in these cells did not affect the cell surface expression of the HTLV-1 receptor Glut1 and Env (S6 Fig), but reduced the Env/Gag co-localization (Fig 10A and 10B and S7 Fig) and the amount of Env in VLPs (Fig 10C). These results further suggest that M-Sec promotes the incorporation of Env into viral particles.

## Discussion

We previously demonstrated that M-Sec mediates efficient cell-to-cell infection of HTLV-1 both in vitro and in vivo, and proposed that the key for the newly-discovered M-Sec function is the accumulation of Gag puncta [16]. In this study, we revealed that M-Sec promotes the accumulation of large Gag puncta through PIP2, which accelerates the co-localization of Gag and Env and thereby the incorporation of Env into viral particles. Thus, M-Sec is an unreported cellular factor that can promote the process of viral particle formation. Because Tax induces the expression of M-Sec in T cells, and HTLV-1-infected T cells constitutively express M-Sec at higher levels than uninfected T cells, it is likely that M-Sec functions in a positive feedback loop that ensures the efficient transmission of HTLV-1.

Tax induces the expression of M-Sec in T cells (S1 Fig). Meanwhile, it has been shown that Tax is expressed in intermittent bursts [27], and that Tax-expressing cells are rarely detected in freshly-isolated peripheral blood T cells and become detectable when they are cultured [26]. Nevertheless, the freshly-isolated HTLV-1-infected CADM1^+^ T cells constitutively expressed M-Sec and 4-1BB at higher levels than uninfected T cells, even before the ex vivo culture (Fig 1B). The constitutive high expression of 4-1BB is likely due to its strong induction by Tax, as observed for the Tax-nucleofected Jurkat cells (S1 Fig) or the ex vivo-cultured HTLV-1-infected CADM1^+^ T cells (Figs 1C and 2A). Meanwhile, the constitutive high expression of M-Sec is likely due to its sustained induction by Tax, as observed for the ex vivo-cultured HTLV-1-infected CADM1^+^ T cells (Fig 2C) or CD4^+^ T cells of HTLV-1^+^ individuals (Fig 2D and 2E).

In the ex vivo culture of infected T cells, M-Sec mRNA was slowly but steadily induced, which was in sharp contrast to the rapid but transient induction of 4-1BB or HIAP1 (Fig 2D). The difference may be related to the differential induction of these genes by the Tax mutants (S1 Fig). The M7 and M22 mutants, both of which are defective in the activation of NF-κB [24], did not induce any of these genes. However, the M47 mutant, which is intact in the activation of NF-κB but defective in the activation of MAP kinases [24] and CREB/ATF transcription factors [43], induced 4-1BB and HIAP1 strongly, but M-Sec modestly. Thus, it is likely that the induction of M-Sec by Tax is more complicated than that of 4-1BB and HIAP1, and dependent on not only NF-κB but also MAP kinases and/or CREB/ATF transcription factors. Such difference may result in the unique induction kinetics of M-Sec in the ex vivo culture (Fig 2D).

The present study suggests that M-Sec promotes the redistribution of PIP2 and thereby promotes the accumulation of HTLV-1 Gag puncta to the PIP2-rich compartments (Figs 5, 6 and 7). The previous studies have suggested that HTLV-1 Gag is less dependent on PIP2 for its puncta formation than HIV-1 Gag [18,19]. This difference may be because HTLV-1 Gag, but not HIV-1 Gag, binds both PIP2 and other acidic lipids such as phosphatidylserine [18,19]. Meanwhile, M-Sec promotes the intracellular accumulation of PIP2. Under such conditions, HTLV-1 Gag can be attracted to the PIP2-rich compartments because the compartments are highly negatively charged. How M-Sec promotes the accumulation of HIV-1 Gag puncta to the PIP2-rich compartments is under investigation.

The M-Sec mutants lacking the PIP2 binding ability failed to promote the accumulation of the large Gag puncta (Fig 5), further supporting the important role of PIP2, presumably by promoting the PIP2 accumulation at intracellular sites that would become the large Gag puncta, as discussed above. A number of cellular proteins have been proposed to bind PIP2 and induce the clustering of PIP2 [37]. However, whether the PIP2 redistribution observed in this study requires PIP2 clustering induced by other proteins remains unexplored. How M-Sec precisely promotes the PIP2 accumulation to intracellular compartments also remains unexplored. M-Sec showed a diffuse localization in SLB-1 and MT-2 cells (S8 Fig), as in other cell types [7,11,14]. When expressed in 293 cells, the wild-type M-Sec, but not M-Sec mutants lacking the PIP2 binding ability (ΔK1 and ΔK2), induced the formation of membrane protrusions and localized at the protrusions (S9 Fig). However, both the wild-type and mutant M-Sec mainly localized throughout the cytoplasm (S9 Fig). Thus, the binding to PIP2 may not be sufficient to explain the M-Sec-mediated PIP2 accumulation. M-Sec is thought to initiate TNT formation through RalA and the exocyst complex [6,7]. Among eight components of the exocyst complex, Sec3 (also known as EXOC1) and Exo70 bind PIP2 [44,45]. Thus, it is possible that M-Sec may promote the PIP2 accumulation, in conjunction with Sec3 and/or Exo70. PIP2 is the well-characterized regulator of actin cytoskeleton, and the actin remodeling can lead to membrane extension/ruffles, filopodia, or lamellipodia [36–38]. Whether host proteins regulating TNT formation or cell motility are involved in the formation of large Gag puncta is under investigation.

It is not fully understood how the incorporation of HTLV-1 Env into viral particles is regulated. One of the possibilities is the incorporation through direct or indirect interaction with Gag. In fact, Blot et al. previously demonstrated that Env and Gag co-localize in HTLV-1-infected activated primary T cells or Jurkat T cell line transfected with the HTLV-1 molecular clone [46]. The present study also supports the Env-Gag interaction, since the cellular distribution of Env is not affected by M-Sec when Gag is absent (S5 Fig), but strongly altered when Gag forms the large cytoplasmic puncta by M-Sec (Fig 8 and S4 Fig). Thus, it is reasonable to speculate that the Gag puncta recruit Env through direct or indirect interaction, which facilitates the Gag/Env co-localization. This idea is consistent with the finding that the amount of Env in VLPs produced by M-Sec-expressing cells is higher than that by the control cells (Figs 9 and 10). Such Gag/Env co-localization may serve as platforms for the formation of virus particles and facilitate the transmission of these particles since it was reported that Gag and Env accumulate at the site of tight cell-to-cell contact and that Gag and Env are rapidly transferred to the target cell [1]. The large Gag puncta formed in the presence of M-Sec localized at intracellular compartments, but not at the plasma membrane (Figs 3 and 4). The small Gag puncta in the absence of M-Sec also localized at intracellular compartments (Figs 3 and 4), as reported previously [18]. Such localization is unique to HTLV-1 Gag because HIV-1 Gag mainly localized at the plasma membrane [18]. Interestingly, despite this difference in subcellular localization, which is determined by the MA domains [18,19], not only HIV-1 Gag but also HTLV-1 Gag and HIV-1 Gag chimera containing HTLV-1 MA efficiently release viral particles [18,19]. These findings are consistent with the idea that the large HTLV-1 Gag puncta that localize at intracellular compartments contribute to the formation of infectious viral particles, although more detailed analyses of HTLV-1 Gag trafficking are required.

The signaling cascades responsible for the M-Sec induction by Tax and the molecular mechanism for the sustained M-Sec expression in the ex vivo-cultured infected T cells are still obscure. It will be necessary to clarify whether M-Sec affects the structures of viral particles. Also, it will be important to clarify how M-Sec utilizes its effectors, such as RalA and the exocyst complex, to promote the redistribution of PIP2 and HTLV-1 Gag. Despite these unresolved questions, M-Sec and its downstream effectors including the exocyst complex are useful to further understand the process of viral particle formation and cell-to-cell infection of HTLV-1.

## Materials and methods

### Ethics statement

PBMCs of asymptomatic HTLV-1 carriers and individuals with HAM/TSP were used. Individuals with HAM/TSP were diagnosed according to the World Health Organization criteria. Written informed consent was obtained from all participants in accordance with the Declaration of Helsinki. All protocols were reviewed and approved by the institutional review boards of Kagoshima University and Kumamoto University.

### Purification of CD4^+^ T cells of HTLV-1-infected individuals, M-Sec qRT-PCR, and Gag immunofluorescence

*Sorting*; The CD3^+^CD4^+^ cells expressing CADM1 were analyzed as HTLV-1-infected cells [25]. To prevent the contamination of monocytes, which highly express M-Sec, CD14 was used as an additional marker [16]. The CD3^+^CD4^+^CD14^-^CADM1^+^ cells in the live cell gate were sorted from PBMCs of asymptomatic HTLV-1 carriers or individuals with HAM/TSP using FACSAria III (BD Biosciences). In selected experiments, the sorted CD3^+^CD4^+^CD14^-^CADM1^+^ cells were cultured with RPMI1640 medium/10% FCS containing 10 ng/mL recombinant human (rh)IL-2 (BioLegend). The uninfected CD3^+^CD4^+^CD14^-^CADM1^-^ cells were sorted as a reference. Monocytes were also sorted as a control of M-Sec-expressing cells. The antibodies used for the sorting were as follows: Pacific Blue-labeled anti-CD3 (OKT3), allophycocyanin (APC)-labeled anti-CD4 (RPA-T4), FITC-labeled CD14 (M5E2; all from BioLegend), and PE-labeled anti-CADM1 (3E1; MBL, Nagoya, Japan).

*M-Sec qRT-PCR*; Total RNA of the sorted cells was isolated using RNeasy micro kit (Qiagen). cDNA was prepared using M-MLV RT (Invitrogen), and real-time RCR was performed using SYBR Premix Ex Taq II (TaKaRa-Bio) and LightCycler (Roche) to quantify the expression level of M-Sec, 4-1BB, and HIAP1. β-actin mRNA was quantified as an internal control. The levels of mRNA expression were calculated using the ΔΔCt method [47]. The primer pairs used were as follows: 5’-CGACACCTACATGCTG-3’ and 5’-CGAGCCCCATACCCTG-3’ (M-Sec), 5’-CTGTTGCTTTGGGACATTTAACGA-3’ and 5’-GGCTGGAGATGGTCCACAGA-3’ (4-1BB), 5’-GACTCAGGTGTTGGGAATCTGGA-3’ and 5’-ACTGGCTTGAACTTGACGGATG-3’ (HIAP1), and 5’-TGACGGGGTCACCCACACTG-3’ and AAGCTGTAGCCGCGCTCGGT-3’ (β-actin).

*Gag immunofluorescence*; The sorted CD3^+^CD4^+^CD14^-^CADM1^+^ cells were resuspended into RPMI1640 medium/10% FCS containing 10 ng/mL rhIL-2, seeded onto collagen-coated chamber slides (Matsunami-glass, Osaka, Japan), cultured in the absence or presence of the M-Sec inhibitor [14,16,29,30]. The M-Sec inhibitor (NPD3064) was identified by an affinity-based chemical array screening [14]. In brief, compounds were arrayed onto the photoaffinity-linker coated slides, which were incubated with the lysates of 293 cells expressing the control DsRed or DsRed-M-Sec fusion protein. The fluorescent signals were quantified, and the identified NPD3064 was synthesized at Pharmeks (Moscow, Russia) or SAI Life Sciences (Telangana, India). The inhibitor was dissolved in DMSO, and added to cultures at a final concentration of 10 μM (0.1% v/v), and the same volume of DMSO was added as a vehicle control. Then, the cells were fixed in 2% paraformaldehyde, permeabilized with 0.2% Triton X-100, and stained with anti-p24 Gag antibodies (6G9; Santa Cruz Biotechnology) followed by AlexaFluor633-labeled anti-mouse IgG (Molecular Probes). Signals were visualized using confocal laser-scanning microscope TCS SP8, and image processing was performed using LAS X software ver.1.4.5 (both from Leica). The signal of Gag was also quantified using ImageJ 1.52n software (NIH).

### Public RNA-Seq data analysis

RNA-Seq data of HTLV-1-infected individuals (two carriers and four individuals with HAM/TSP), which are publicly available in the NCBI Gene Expression Omnibus (#GSE234450) [28], were downloaded and analyzed for the mRNA expression of M-Sec, 4-1BB, or HIAP1, using iDEP (v0.96) software [48].

### Tax nucleofection into Jurkat cells

Jurkat cells (clone E6-1) obtained from the American Tissue Culture Collection were maintained with RPMI1640 medium/10% FCS, and nucleofected with the Tax plasmid using Nucleofector II (the program T-014) and Cell line Nucleofector kit V (both from Lonza). The wild-type, M7, M22, and M47 mutant Tax plasmids cloned into pGFP-C1 (Clontech) were provided by C.R.M. Bangham (Imperial College London, United Kingdom). The nucleofected cells were subjected to qRT-PCR for M-Sec mRNA as described previously [16]. Alternatively, in order to confirm the comparable expression of GFP-fused Tax protein, the cells were analyzed for GFP expression by flow cytometry, using CytoFLEX (Beckman Coulter) and FlowJo software (FlowJo LLC).

### SLB-1 and MT-2 cells

SLB-1 cells were provided by M. Fujii (Niigata University, Japan). MT-2 cells were obtained from the Japanese Collection of Research Bioresources Cell Bank. These cells were maintained with RPMI1640 medium/10% FCS. The knockdown of M-Sec in these cells were performed using MISSION shRNA lentiviral transduction particles (Sigma), as described previously [16].

### 293 cells stably expressing M-Sec

The 293 cells (Invitrogen) were maintained with DMEM medium/10% FCS, and engineered to express human M-Sec using the lentiviral system. The human M-Sec cDNA (NCBI GenBank #M92357.1) was synthesized at Eurofins Japan and cloned into the lentiviral vector pLVSIN-EF1α-Hyg (TaKaRa-Bio). The lentivirus was prepared and added to 293 cells, and cells expressing human M-Sec were selected under the cultures containing 200 μg/ml hygromycin B. The 293 cells were also engineered to express GFP-fused mouse M-Sec. They were transfected with the plasmid encoding the wild-type or mutant (ΔK1 or ΔK2) [7], using Lipofectamine 3000 reagent (Invitrogen). (see below for details). The cells expressing GFP-fused M-Sec were selected under the cultures containing 1,200 μg/ml G418, and enriched by cell sorting using FACSAria II.

### Transfection using 293 cells

The 293 cells were seeded onto 12-wll plates (1.8 x 10^5^ cells/well), cultured, and transfected with various plasmids using 3 μl Lipofectamine 3000 reagent and 2 μl P3000 reagent (both from Invitrogen). The total amount of the plasmid (1 μg) was normalized using appropriate control (empty) vectors. After 6 h of transfection, the culture medium was replaced with fresh medium. The cells were further cultured and subjected to immunofluorescence, live cell imaging, western blotting, flow cytometry, or ELISA.

### Plasmids

The plasmids used for transient transfection into 293 cells were as follows: the molecular clone pX1MT-M [31], the Gag expression plasmid pCRVI/HTLV-1/Gag-Flag [35], the Env expression plasmid pcDNA-1E-RRE [35], the Rev expression plasmid pRSV-Rev (provided by N.R. Landau, New York University Grossman School of Medicine, United States) [49], the expression plasmids for the wild-type or Δ1 mutant of 5ptaseIV (pcDNA4TO/Myc5ptaseIV-WT or pcDNA4TO/Myc5ptaseIV-Δ1) [40], and the GFP-PLCδ-PH expression plasmid (#21179; Addgene) [42]. The HA-tagged Gag expression plasmid pCRVI/HTLV-1/Gag-HA [35] was used for the co-staining of Gag and Myc-tagged 5ptaseIV (Fig 6C).

### Immunofluorescence and live cell imaging

Immunofluorescence was performed as described previously [16]. In brief, cells were fixed in paraformaldehyde, permeabilized with Triton X-100, and stained with anti-p24 Gag (6G9; Santa Cruz Biotechnology) or anti-Env antibodies (LAT-27) [50] followed by AlexaFluor568-labeled anti-mouse IgG, AlexaFluor488-labeled anti-mouse IgG, or AlexaFluor568-labeled anti-rat IgG antibodies (all from Molecular Probes). Cells were also stained with phalloidin conjugated to AlexaFluor488 and DAPI (both from Molecular Probes) to visualize F-actin and nuclei, respectively. For the co-staining of Gag and 5ptaseIV (Fig 6C), cells were stained with anti-HA (C29F4; Cell Signaling Technology) (to detect HA-tagged Gag) and anti-Myc antibodies (9E10; Santa Cruz Biotechnology) (to detect Myc-tagged 5ptaseIV) followed by AlexaFluor568-labeled anti-rabbit IgG and AlexaFluor488-labeled anti-mouse IgG (both from Molecular Probes). Cells were also stained with phalloidin conjugated to AlexaFluor405 (Molecular Probes). Signals were visualized using confocal laser-scanning microscope FV3000 (Olympus). Image processing was performed using FV31S-SW software (Olympus). The size of Gag puncta or membrane protrusions, or cell surface area was measured using ImageJ 1.52n software. Pearson’s correlation coefficients of Gag and Env were calculated using the Coloc2 plugin in the ImageJ software program. To visualize PIP2 in living cells, 293 cells were transfected with the GFP-PLCδ-PH plasmid and analyzed using the confocal laser-scanning microscope TCS SP8.

### Western blotting

Western blotting was performed as described previously [16]. In brief, cells were lysed with Nonidet P-40 lysis buffer containing protease- and phosphatase inhibitors. The total cell lysates were subjected to western blotting. The antibodies used were as follows: anti-M-Sec (F-6; Santa Cruz Biotechnology) (to detect human M-Sec), anti-M-Sec (H-654; Santa Cruz Biotechnology) (to detect mouse M-Sec), anti-p24 Gag (6G9; Santa Cruz Biotechnology), anti-p19 Gag (TP-8; ZeptoMetrix), anti-Env (clone D) [51], anti-Myc (to detect Myc-tagged 5ptaseIV) (9E10; Sigma), anti-actin (EPR16769; Abcam), and anti-GAPDH (0411; Santa Cruz Biotechnology). Detection was performed using HRP-labeled secondary antibodies (GE Healthcare), western blot ultra-sensitive HRP substrate (TaKaRa-Bio) or ECL select western blotting detection reagent (Cytiva), and ImageQuant LAS4000 image analyzer (GE Healthcare) or Amersham ImageQuant 800 (Cytiva).

### Flow cytometry

The 293 cells transfected with the GFP-PLCδ-PH expression plasmid were analyzed for GFP expression by flow cytometry, as described above. In order to detect dead cells, the cells were also stained with Fixable near-IR dead cell stain kit (for 633 or 635 nm excitation; ThermoFisher). SLB-1 cells were analyzed for the cell surface expression of Glut1 or Env by flow cytometry, using APC-labeled anti-Glut1antibodies (202915; R&D Systems) or anti-Env antibodies (LAT-27) [50] followed by APC-labeled anti-rat IgG antibodies (BD Biosciences).

### Viral production

To monitor the viral production, the supernatants of transfected 293 cells were clarified by centrifugation and analyzed for their p19 Gag concentrations by ELISA (ZeptoMetrix).

### Analyses of VLPs

VLPs in the supernatants of transfected 293 cells were collected by centrifugation at 20,000 x g for 60 min at 4°C after filtration using 0.45 μm PVDF filter [52]. The pellets were dissolved in SDS-PAGE sample buffer and subjected to western blotting as described above. Total cell lysates were also prepared and analyzed by western blotting to verify the expression of Gag or Env.

### Statistical analysis

Differences between two groups were analyzed by unpaired Student’s *t*-test. Differences between three groups were analyzed by one-way ANOVA. Pearson correlation analysis was also performed. All statistical analyses were conducted using PRISM 10 (GraphPad).

## Supporting information

S1 Fig Induction of M-Sec expression by Tax in Jurkat cells. (**A**) Jurkat cells were nucleofected with the empty vector (-) (1 μg), or GFP-fused Tax plasmid expressing the wild-type (WT) or the indicated mutant (3 μg). After 24 h, the cells were analyzed for the expression of M-Sec, 4-1BB, or HIAP1 mRNA by qRT-PCR (n = 3). The expression level shown is relative to that of the empty vector-nucleofected control cells. (**B**) Jurkat cells were left untreated (-), or nucleofected with the indicated Tax expression plasmid (3 μg). After 24 h, the cells were analyzed for the GFP expression by flow cytometry (to detect GFP-fused Tax protein). The percentage of GFP^+^ cells is shown.
(TIF)

S2 Fig Effect of M-Sec inhibitor on Gag puncta in CD4^+^ T cells of an individual with HAM/TSP. (**A**, **B**) The CD3^+^CD4^+^CADM1^+^ cells were sorted from PBMCs of an individual with HAM/TSP, cultured with DMSO (vehicle) or 10 μM M-Sec inhibitor [14] for 48 h, and analyzed for Gag (red) by immunofluorescence. The nuclei were stained with DAPI (blue). The images shown are the overlay composed of ten serial Z-sections (two cells for each group). Scale bar: 4.15 μm. In **B**, the immunofluorescence images were scanned and quantified for the signal of Gag (two cells for each group). In upper panels, the position of the accumulated Gag puncta is indicated by orange arrowheads. In **A** and **B**, the typical images are shown.
(TIF)

S3 Fig Size of membrane protrusions and cell surface area of M-Sec-expressing 293 cells. The control (Cr) 293 cells or 293 cells stably expressing M-Sec were stained with phalloidin (to visualize F-actin) and analyzed for the average size of membrane protrusions in each cell (upper panel, 25 cells for each group) or the cell surface area (lower panel, 30 cells for each group). *p < 0.05.
(TIF)

S4 Fig Localization of Env and Gag in M-Sec-expressing 293 cells transfected with molecular clone. (**A**, **B**) The control (Cr) 293 cells or 293 cells stably expressing M-Sec were transfected with the HTLV-1 molecular clone pX1MT-M (1 μg), cultured for 2 days, and analyzed for Env (red) or Gag (green) by immunofluorescence. In middle panels, the nuclei were stained with DAPI (blue). In right panels, the magnified images of "a" and "b" in the left panels are shown. Scale bar: 10 μm. In **B**, Pearson’s correlation coefficients between Gag and Env are shown. *p < 0.05.
(TIF)

S5 Fig Localization of Env in M-Sec-expressing 293 cells transfected with Env expression plasmid. (**A**) The control (Cr) 293 cells or 293 cells stably expressing M-Sec were co-transfected with the Env expression plasmid pcDNA-1E-RRE (0.3 μg) and the Rev expression plasmid pRSV-Rev (0.4 μg), cultured for 2 days, and analyzed for Env (red) by immunofluorescence. In right panels, the nuclei and F-actin were stained with DAPI (blue) and phalloidin (green), respectively. Scale bar: 10 μm. (**B**) Cr-293 cells and M-Sec-293 cells were transfected and cultured as in **A**, and analyzed for the expression of Env by western blotting. GAPDH blot is the loading control.
(TIF)

S6 Fig Effect of M-Sec knockdown on cell surface expression of Glut1 and Env in SLB-1 cells. The control (Cr) SLB-1 cells or M-Sec knockdown (ΔM-Sec) SLB-1 cells were analyzed for the cell surface expression of Glut1 (upper panel) or Env (lower panel) by flow cytometry (n = 3). The expression level shown is relative to that of Cr SLB-1 cells. *n*.*s*., not significant.
(TIF)

S7 Fig Effect of M-Sec knockdown on Env/Gag co-localization in MT-2 cells. The control (Cr) MT-2 cells or M-Sec knockdown (ΔM-Sec) MT-2 cells were analyzed for Gag (green), Env (red) or F-actin (blue) by immunofluorescence. In right panels, the magnified images of "a", "b", "c" and "d" in the left and middle panels are shown. Scale bar: 10 μm (left and middle panels) or 5 μm (right panels).
(TIF)

S8 Fig Localization of M-Sec in SLB-1 and MT-2 cells. SLB-1 or MT-2 cells were analyzed for M-Sec (green) by immunofluorescence. The nuclei and F-actin were stained with DAPI (blue) and phalloidin (red), respectively. Scale bar: 10 μm.
(TIF)

S9 Fig Localization of M-Sec in transfected 293 cells. The control 293 cells were transfected with the M-Sec plasmid encoding the wild-type (WT) or mutant (ΔK1 or ΔK2). The cells were cultured for 2 days, and analyzed for the localization of the GFP-fused M-Sec by live cell imaging. Scale bar: 10 μm.
(TIF)
