# Supplementary figures and images for: M-Sec promotes the accumulation of intracellular HTLV-1 Gag puncta and the incorporation of Env into viral particles
Source: PLoS Pathog. 2025 Jan 27;21(1):e1012919. doi: 10.1371/journal.ppat.1012919 (PMC11801699; doi:10.1371/journal.ppat.1012919)

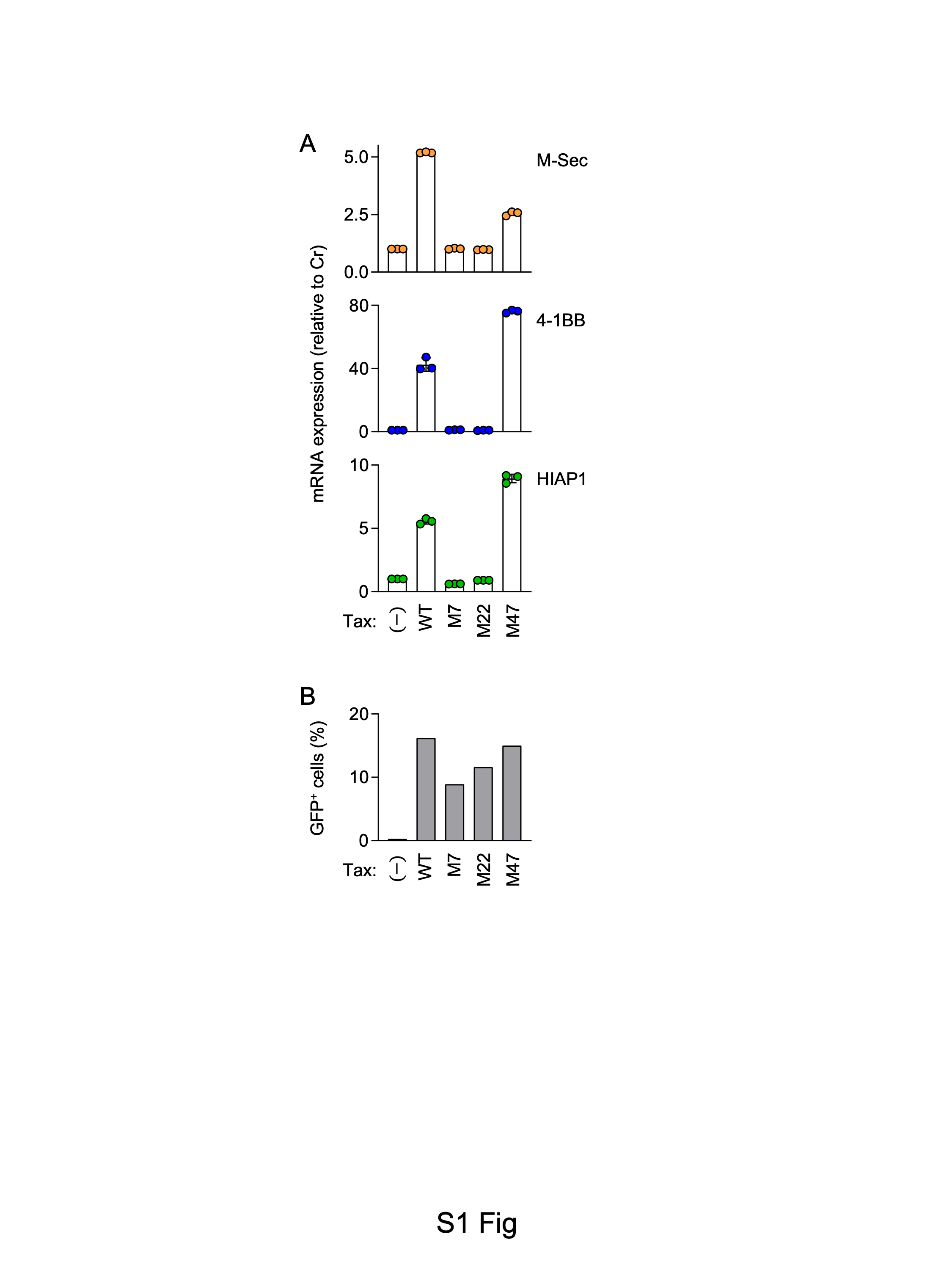

Supplement: S1 Fig — (A) Jurkat cells were nucleofected with the empty vector (-) (1 μg), or GFP-fused Tax plasmid expressing the wild-type (WT) or the indicated mutant (3 μg). After 24 h, the cells were analyzed for the expression of M-Sec, 4-1BB, or HIAP1 mRNA by qRT-PCR (n = 3). The expression level shown is relative to that of the empty vector-nucleofected control cells. (B) Jurkat cells were left untreated (-), or nucleofected with the indicated Tax expression plasmid (3 μg). After 24 h, the cells were analyzed for the GFP expression by flow cytometry (to detect GFP-fused Tax protein). The percentage of GFP+ cells is shown. (TIF) [file ppat.1012919.s001.tif]

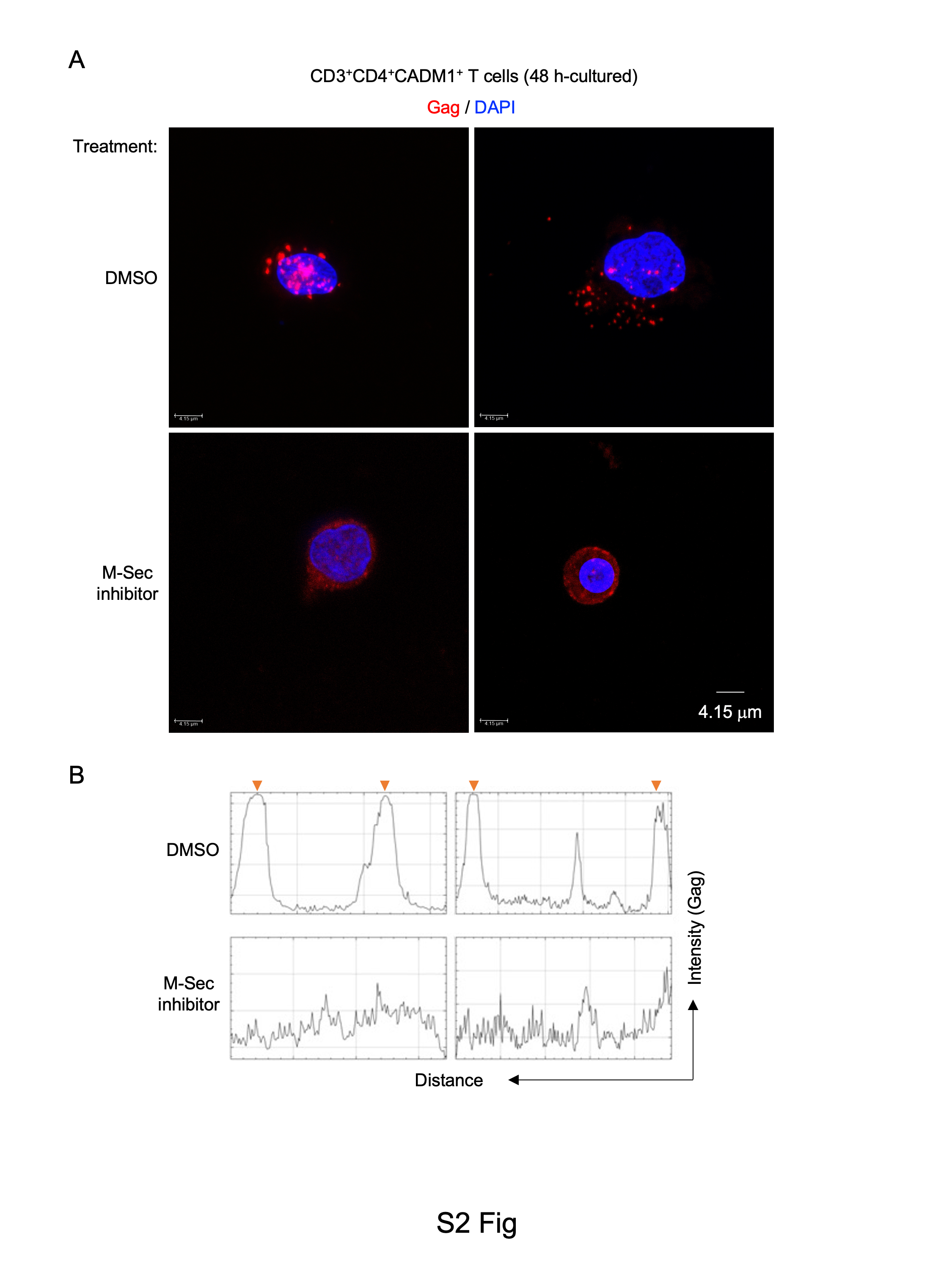

Supplement: S2 Fig — (A, B) The CD3+CD4+CADM1+ cells were sorted from PBMCs of an individual with HAM/TSP, cultured with DMSO (vehicle) or 10 μM M-Sec inhibitor [14] for 48 h, and analyzed for Gag (red) by immunofluorescence. The nuclei were stained with DAPI (blue). The images shown are the overlay composed of ten serial Z-sections (two cells for each group). Scale bar: 4.15 μm. In B, the immunofluorescence images were scanned and quantified for the signal of Gag (two cells for each group). In upper panels, the position of the accumulated Gag puncta is indicated by orange arrowheads. In A and B, the typical images are shown. (TIF) [file ppat.1012919.s002.tif]

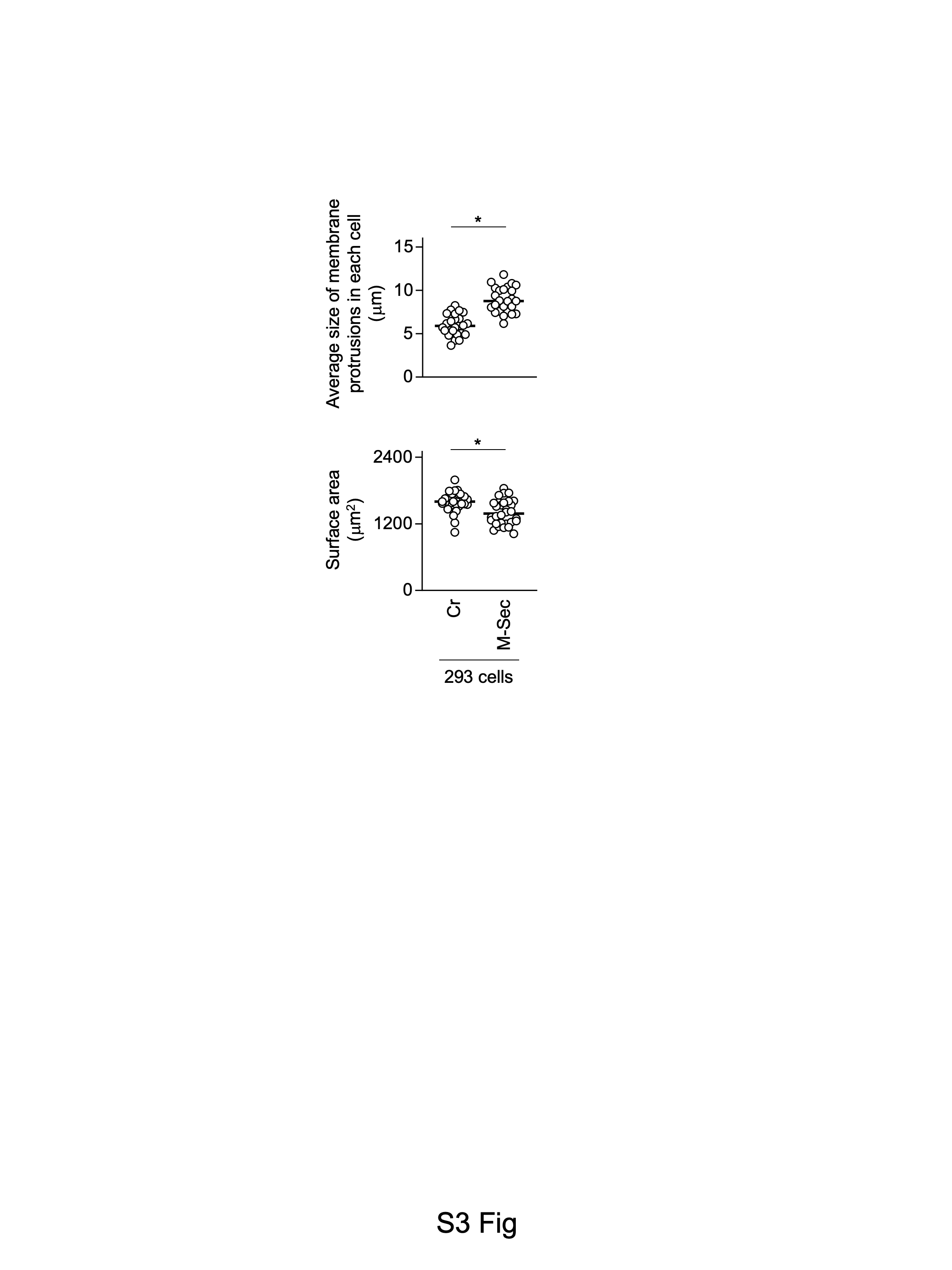

Supplement: S3 Fig — The control (Cr) 293 cells or 293 cells stably expressing M-Sec were stained with phalloidin (to visualize F-actin) and analyzed for the average size of membrane protrusions in each cell (upper panel, 25 cells for each group) or the cell surface area (lower panel, 30 cells for each group). *p < 0.05. (TIF) [file ppat.1012919.s003.tif]

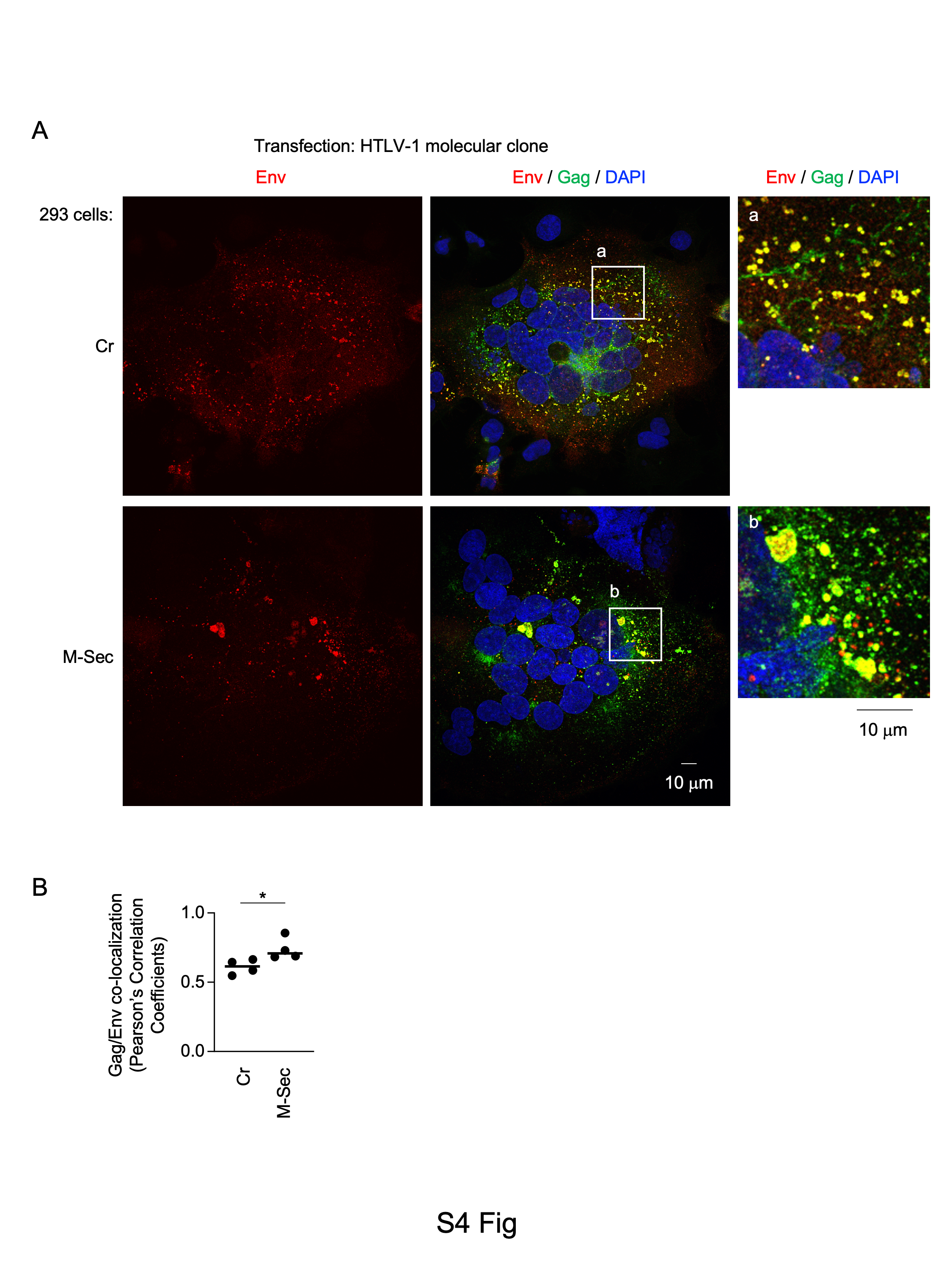

Supplement: S4 Fig — (A, B) The control (Cr) 293 cells or 293 cells stably expressing M-Sec were transfected with the HTLV-1 molecular clone pX1MT-M (1 μg), cultured for 2 days, and analyzed for Env (red) or Gag (green) by immunofluorescence. In middle panels, the nuclei were stained with DAPI (blue). In right panels, the magnified images of "a" and "b" in the left panels are shown. Scale bar: 10 μm. In B, Pearson’s correlation coefficients between Gag and Env are shown. *p < 0.05. (TIF) [file ppat.1012919.s004.tif]

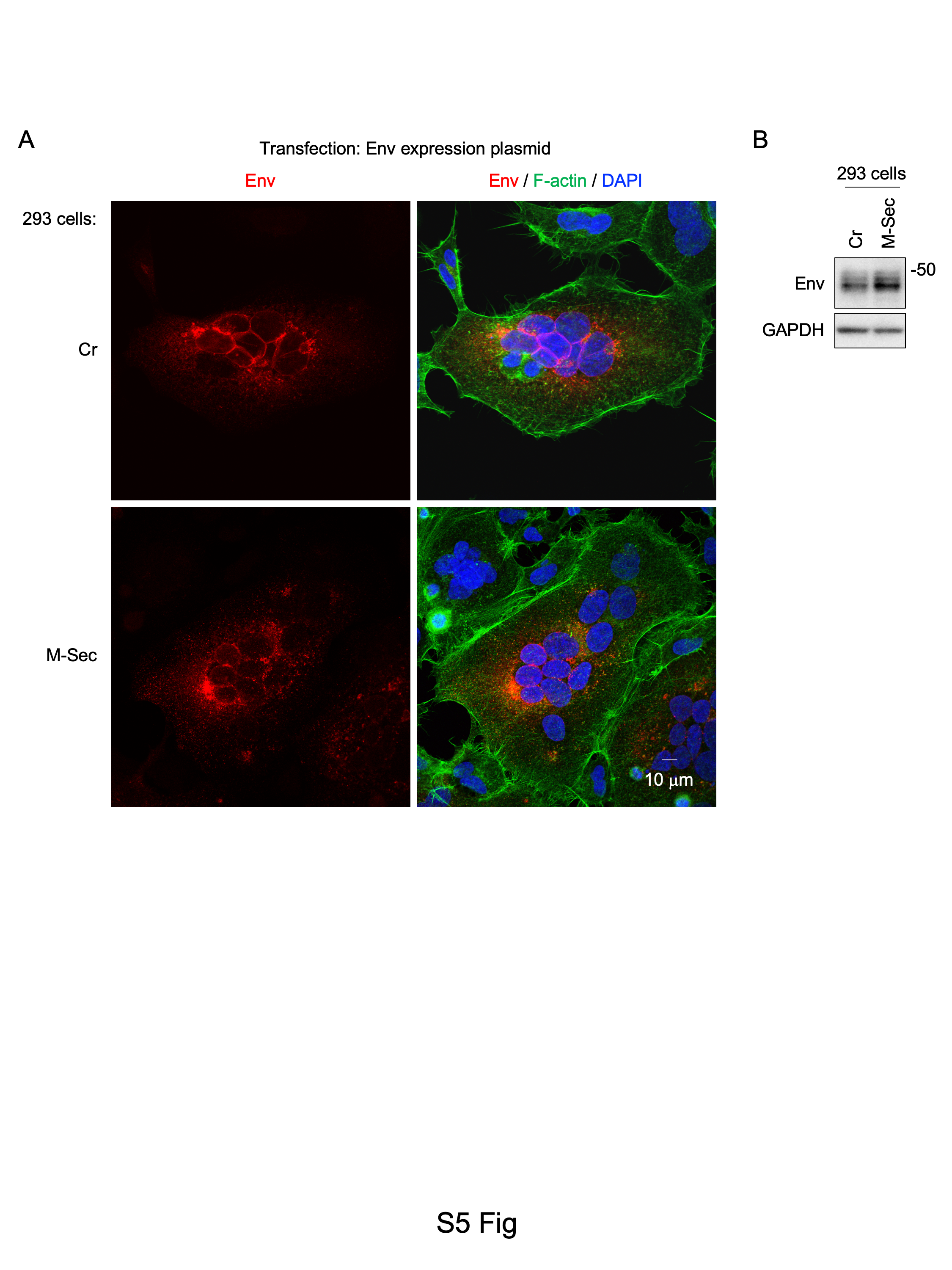

Supplement: S5 Fig — (A) The control (Cr) 293 cells or 293 cells stably expressing M-Sec were co-transfected with the Env expression plasmid pcDNA-1E-RRE (0.3 μg) and the Rev expression plasmid pRSV-Rev (0.4 μg), cultured for 2 days, and analyzed for Env (red) by immunofluorescence. In right panels, the nuclei and F-actin were stained with DAPI (blue) and phalloidin (green), respectively. Scale bar: 10 μm. (B) Cr-293 cells and M-Sec-293 cells were transfected and cultured as in A, and analyzed for the expression of Env by western blotting. GAPDH blot is the loading control. (TIF) [file ppat.1012919.s005.tif]

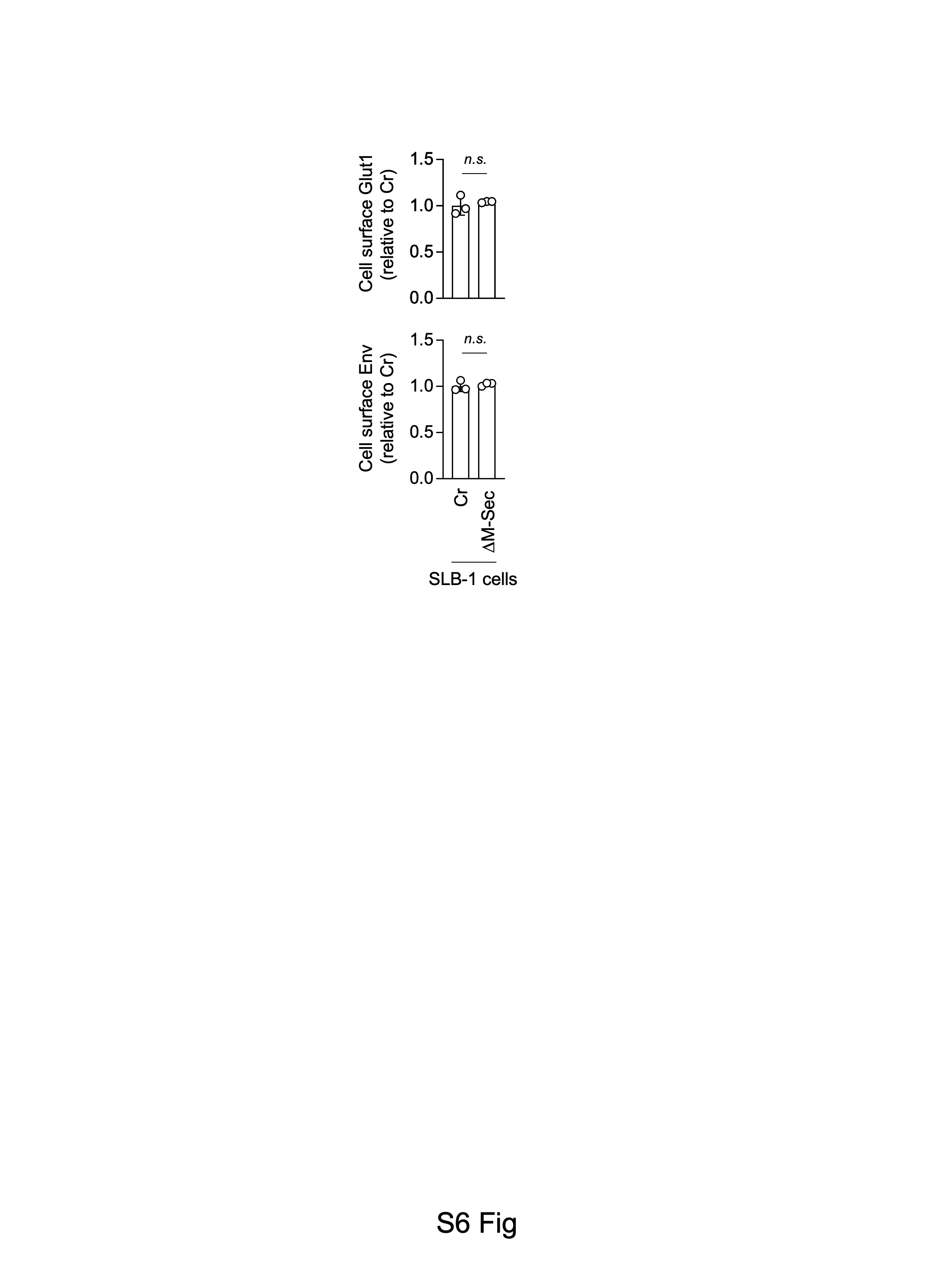

Supplement: S6 Fig — The control (Cr) SLB-1 cells or M-Sec knockdown (ΔM-Sec) SLB-1 cells were analyzed for the cell surface expression of Glut1 (upper panel) or Env (lower panel) by flow cytometry (n = 3). The expression level shown is relative to that of Cr SLB-1 cells. n.s., not significant. (TIF) [file ppat.1012919.s006.tif]

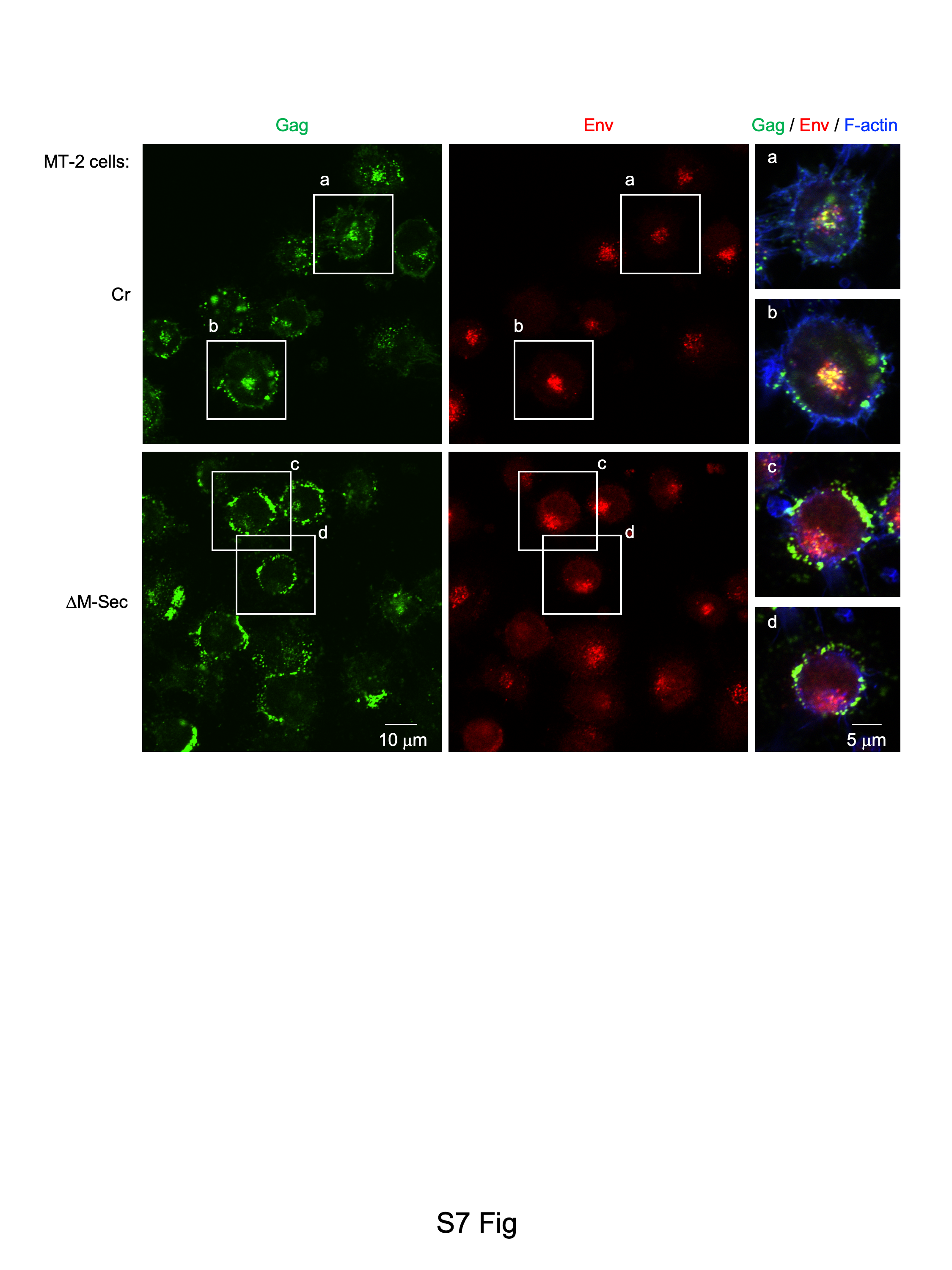

Supplement: S7 Fig — The control (Cr) MT-2 cells or M-Sec knockdown (ΔM-Sec) MT-2 cells were analyzed for Gag (green), Env (red) or F-actin (blue) by immunofluorescence. In right panels, the magnified images of "a", "b", "c" and "d" in the left and middle panels are shown. Scale bar: 10 μm (left and middle panels) or 5 μm (right panels). (TIF) [file ppat.1012919.s007.tif]

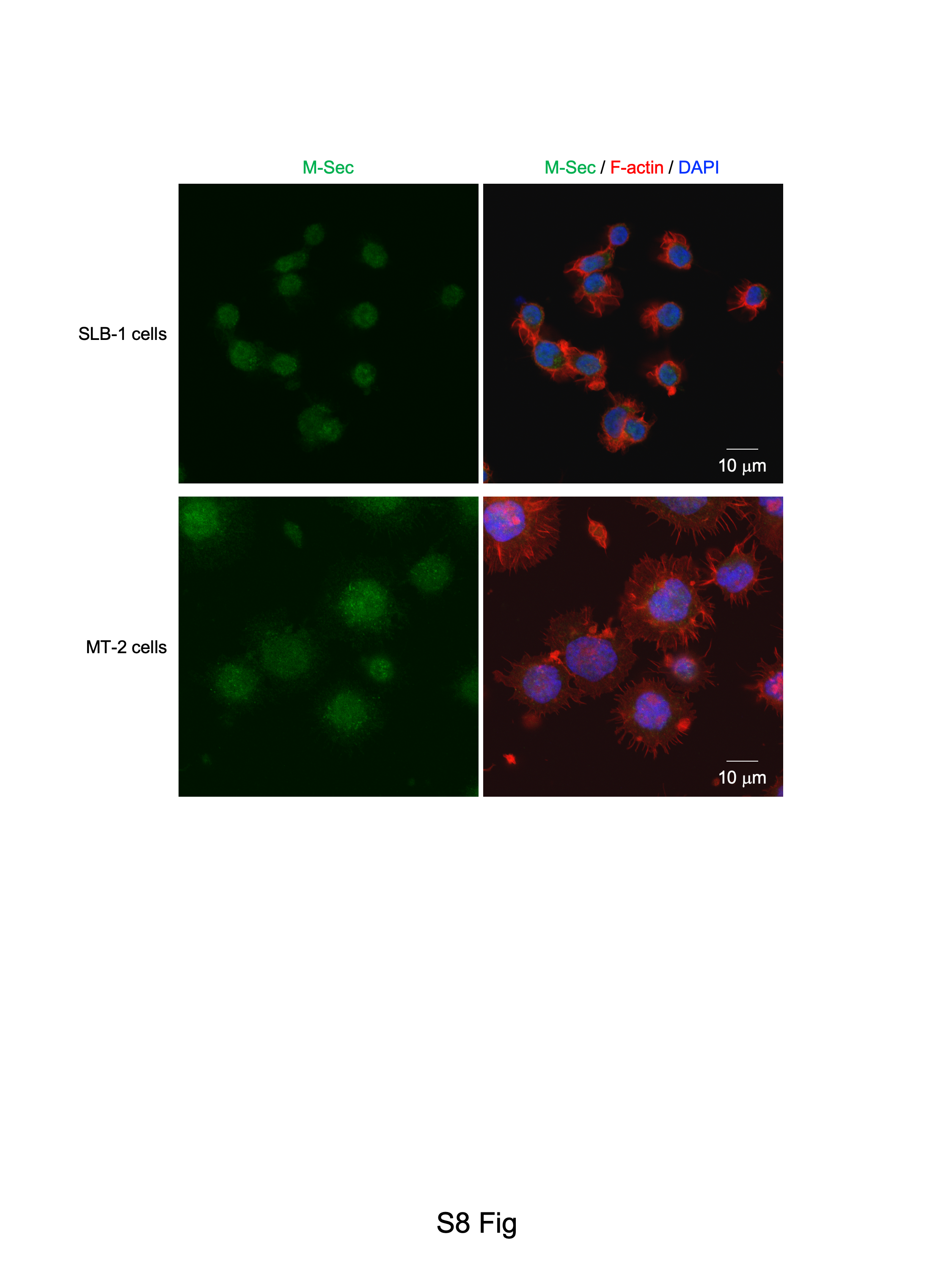

Supplement: S8 Fig — SLB-1 or MT-2 cells were analyzed for M-Sec (green) by immunofluorescence. The nuclei and F-actin were stained with DAPI (blue) and phalloidin (red), respectively. Scale bar: 10 μm. (TIF) [file ppat.1012919.s008.tif]

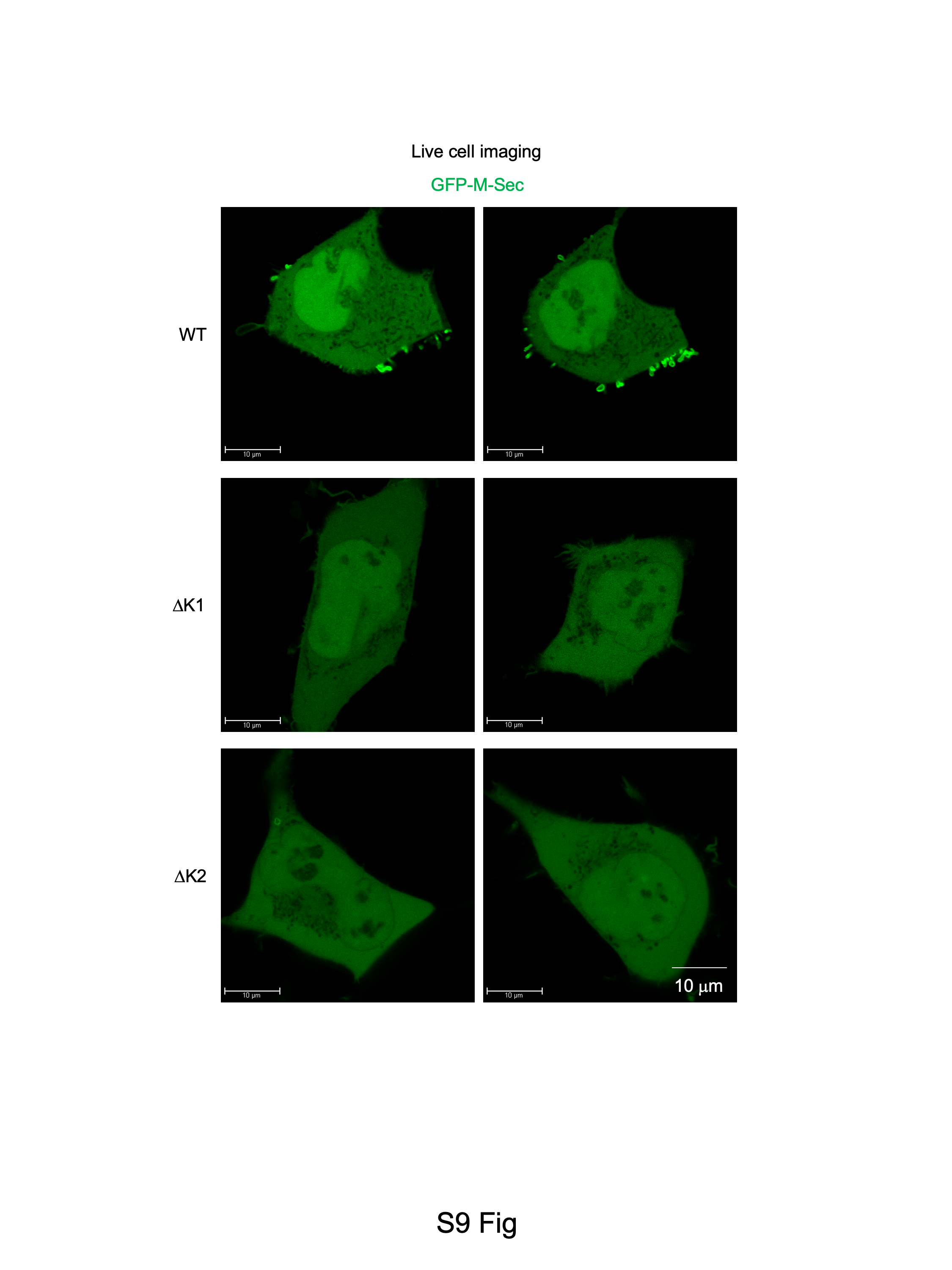

Supplement: S9 Fig — The control 293 cells were transfected with the M-Sec plasmid encoding the wild-type (WT) or mutant (ΔK1 or ΔK2). The cells were cultured for 2 days, and analyzed for the localization of the GFP-fused M-Sec by live cell imaging. Scale bar: 10 μm. (TIF) [file ppat.1012919.s009.tif]
