# Supplementary material for: M-Sec promotes the accumulation of intracellular HTLV-1 Gag puncta and the incorporation of Env into viral particles
Source: PLoS Pathog. 2025 Jan 27;21(1):e1012919. doi: 10.1371/journal.ppat.1012919 (PMC11801699; doi:10.1371/journal.ppat.1012919)
Supplement: Attachment — Submitted filename: Response to Reviewers (R1).pdf [file ppat.1012919.s010.pdf]

<u>Reviewer #1</u>

Part I - Summary

Hiyoshi et al., previously reported that M-Sec, a cellular protein involved in the formation of tunneling nanotubes, is overexpressed in HTLV-1 infected cells. They reported that the viral protein Tax activates the transcription of M-Sec, and that M-Sec promotes HTLV-1 infection through increase in membrane protrusions and cell motility. In this manuscript, "M-Sec promotes the accumulation of intracellular HTLV-1 Gag puncta and the incorporation of Env into viral particles", the same authors further investigate how M-Sec could stimulate HTLV-1 infection. They show that M-Sec interacts with phosphatidylinositol 4,5-biphosphate (PIP2), through a lysine-rich motif, which promotes the formation of Gag puncta in the cells. They then found that these Gag aggregates lead to Env accumulation, which consequently increased incorporation of Env into viral particles. This is a very interesting study that increases our knowledge of cellular proteins important for HTLV-1 virion formation. Some additional experiments, listed below, are necessary to strengthen the conclusion of the manuscript.

Part II - Major Issues

1. *One major concern is that the authors exclusively use 293 cells to analyze the co-localization of PIP2/Gag/Env in presence or absence of M-Sec. The conclusion of the manuscript will be stronger/more pertinent if some microscopy experiments are also performed in HTLV-1 infected T-cells before and after knock-down of M-Sec.*
2. *Same concern for figure 9, knock-down of M-Sec should be performed in HTLV-1 infected cells to evaluate its effect on Env incorporation into virions.*

Reply to comments-1 and 2

According to the suggestions, we performed additional experiments using HTLV-1-infected M-Sec$^+$ T cell lines (SLB-1 and MT-2), and confirmed that the M-Sec knockdown in these cells reduced the Env/Gag co-localization and the amount of Env in virus-like particles, as shown in the new figures (Fig 10 and S7 Fig). To explain the results and experimental procedures, we added the following sentences (underlined):

[Page 12, lines 3 - 9]

<u>Finally, we performed experiments using HTLV-1-infected M-Sec$^+$ T cell lines, SLB-1 and MT-2. We previously reported that the knockdown of M-Sec in these cells altered the intracellular localization of Gag [16]. In this study, we found that the M-Sec knockdown in these cells did not affect the cell surface expression of the HTLV-1 receptor Glut1 and Env (S6 Fig), but reduced the Env/Gag co-localization (Fig 10A, Fig 10B, S7 Fig) and the amount of Env in VLPs (Fig 10C). These results further suggest that M-Sec promotes</u>

the incorporation of Env into viral particles.

[Page 19, lines 11 - 16 (Materials and methods)]

SLB-1 and MT-2 cells

SLB-1 cells were provided by M. Fujii (Niigata University, Japan). MT-2 cells were obtained from the Japanese Collection of Research Bioresources Cell Bank. These cells were maintained with RPMI1640 medium/10% FCS. The knockdown of M-Sec in these cells were performed using MISSION shRNA lentiviral transduction particles (Sigma), as described previously [16].

[The legend of Fig 10]

Fig 10. Effect of M-Sec knockdown on Env/Gag co-localization and Env incorporation into virus-like particles in SLB-1 and MT-2 cells.

(A) The control (Cr) SLB-1 cells or M-Sec knockdown (ΔM-Sec) SLB-1 cells were analyzed for Gag (green), Env (red) or F-actin (blue) by immunofluorescence. In right panels, the magnified images of "a", "b", "c" and "d" in the left and middle panels are shown. Scale bar: 10 μm (left and middle panels) or 5 μm (right panels).

(B) Pearson's correlation coefficients between Gag and Env are shown. In the left panel, the control (Cr) SLB-1 cells and M-Sec knockdown (ΔM-Sec) SLB-1 cells were analyzed. In the right panel, the control (Cr) MT-2 cells and M-Sec knockdown (ΔM-Sec) MT-2 cells were analyzed (see S7 Fig for immunofluorescence). $^{*}p < 0.05$.

(C) The virus-like particles (VLPs) in the supernatants were collected and analyzed for the amount of Env by western blotting after normalization of the Gag amount. In left blots, the control (Cr) SLB-1 cells and M-Sec knockdown (ΔM-Sec) SLB-1 cells were analyzed. In right blots, the control (Cr) MT-2 cells and M-Sec knockdown (ΔM-Sec) MT-2 cells were analyzed.

[The legend of S7 Fig]

S5 Fig. Effect of M-Sec knockdown on Env/Gag co-localization in MT-2 cells.

The control (Cr) MT-2 cells or M-Sec knockdown (ΔM-Sec) MT-2 cells were analyzed for Gag (green), Env (red) or F-actin (blue) by immunofluorescence. In right panels, the magnified images of "a", "b", "c" and "d" in the left and middle panels are shown. Scale bar: 10 μm (left and middle panels) or 5 μm (right panels).

3. *Another concern is that M-Sec localization in HTLV-1 infected T- cells is never shown. It will be interesting to determine its localization and whether it colocalizes with Gag/Env in these cells. The authors used an M-Sec antibody for immunofluorescence (F-6; Santa Cruz Biotechnology) in a previous publication regarding the role of M-Sec in HIV-1 infection.*

Reply

According to the suggestion, we showed the localization of M-Sec in SLB-1 and MT-2 cells (S8 Fig). M-Sec exhibited a diffuse localization (S8 Fig), the distribution of which was distinct from that of Gag or Env (Fig 10A, S7 Fig). The diffuse localization of M-Sec was also reported in monocyte-derived macrophages [reference 14], nasopharyngeal carcinoma cells [reference 11] and HeLa cells transfected with the M-Sec expression plasmid [reference 7]. When expressed in 293 cells, the wild-type M-Sec, but not M-Sec mutants lacking the PIP2 binding ability (ΔK1 and ΔK2), induced the formation of membrane protrusions and localized at the protrusions (S9 Fig). However, both the wild-type and mutant M-Sec mainly localized throughout the cytoplasm (S9 Fig). These results are consistent with the idea that M-Sec promotes the PIP2 accumulation and thereby Gag puncta accumulation. However, once again, how the diffusely localized M-Sec can induce the PIP2 accumulation is unclear. The binding to PIP2 may not be sufficient to explain the M-Sec-mediated PIP2 accumulation. To discuss these points and explain the results of M-Sec localization, we added the following sentences (underlined):

[Page 14, line 23 - page 15, line 3 (Discussion)]

How M-Sec precisely promotes the PIP2 accumulation to intracellular compartments also remains unexplored. <u>M-Sec showed a diffuse localization in SLB-1 and MT-2 cells (S8 Fig), as in other cell types [7, 11, 14]. When expressed in 293 cells, the wild-type M-Sec, but not M-Sec mutants lacking the PIP2 binding ability (ΔK1 and ΔK2), induced the formation of membrane protrusions and localized at the protrusions (S9 Fig). However, both the wild-type and mutant M-Sec mainly localized throughout the cytoplasm (S9 Fig). Thus, the binding to PIP2 may not be sufficient to explain the M-Sec-mediated PIP2 accumulation.</u>

[The legend of S8 Fig]

<u>S8 Fig. Localization of M-Sec in SLB-1 and MT-2 cells.</u>

<u>SLB-1 or MT-2 cells were analyzed for M-Sec (green) by immunofluorescence. The nuclei and F-actin were stained with DAPI (blue) and phalloidin (red), respectively. Scale bar: 10 μm.</u>

[The legend of S9 Fig]

S9 Fig. Localization of M-Sec in transfected 293 cells.

The control 293 cells were transfected with the M-Sec plasmid encoding the wild-type (WT) or mutant (ΔK1 or ΔK2). The cells were cultured for 2 days, and analyzed for the localization of the GFP-fused M-Sec by live cell imaging. Scale bar: 10 μm.

4. Figure 2D should also show Tax mRNA expression (available in the data set).

Reply

We thank for the comment. In Fig 2D, the RNA-Seq data deposited by Aristodemou et al. [reference 28] were analyzed. In the same study, Aristodemou et al. also performed the time-course qRT-PCR analysis and demonstrated that Tax upregulated as early as 1 day after the ex vivo culture but declined thereafter, as shown below (right panel, modified from S1 Fig of Aristodemou et al., PLoS Pathog 2023). The expression kinetics of Tax was similar to that of 4-1BB and HIAP1, but distinct from that of M-Sec (Fig 2D). To explain these findings, we modified the text as follows (underlined):

[Page 7, lines 10 - 14 in the original manuscript]

When their data deposited in the public NCBI Gene Expression Omnibus (#GSE234450) were analyzed, M-Sec mRNA was slowly but steadily upregulated at least up to day 6, the kinetics of which was distinct from that of 4-1BB or HIAP1 (Fig 2D). These results suggest that HTLV-1 infection causes a sustained expression of M-Sec in $CD4^+$ T cells.

[Page 7, lines 10 - 17 in the revised manuscript]

When their data deposited in the public NCBI Gene Expression Omnibus (#GSE234450) were analyzed, 4-1BB and HIAP were upregulated as early as 1 day after the ex vivo culture but declined thereafter (Fig 2D), the kinetics of which was similar to that of the expression of Tax mRNA [28]. In contrast, M-Sec mRNA was slowly but steadily upregulated at least up to day 6 (Fig 2D). Consistent with this, M-Sec protein was steadily upregulated at least up to day 6 (Fig 2E). These results suggest that HTLV-1 infection causes a sustained expression of M-Sec in $CD4^+$ T cells.

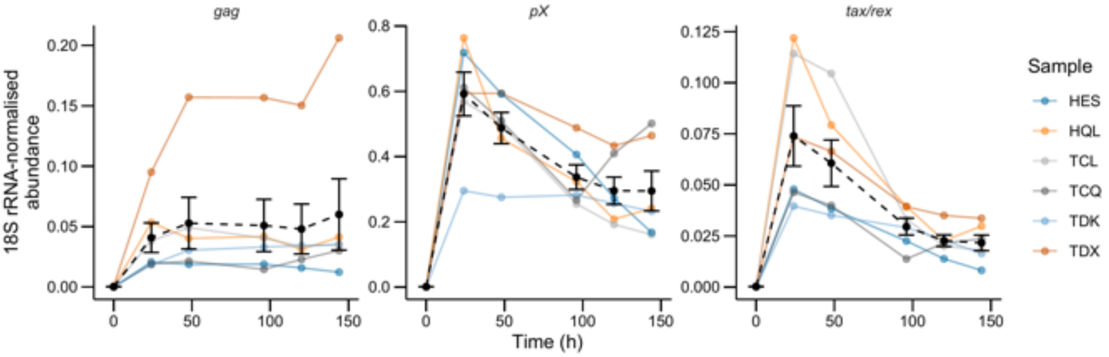

S1 Fig. Proviral transcript expression following reactivation (Aristodemou et al., PLoS Pathog 2023) HTLV-1 sense-strand RNA trajectories during ex vivo culture, obtained using qRT-PCR. ”gag” amplicons correspond to proviral region 2,017–2,203, whilst ”pX” corresponds to region 8,000–8,161. tax/rex amplicons straddle the second exon junction. Coordinates presented for HTLV-1 sequence AB513134. Dark points and dotted lines represent mean ± SEM.

Part III - Minor Issues

1. Figure 4: quantification is missing.

Reply

According to the suggestion, we added the summary of quantification in Fig 4B in the revised manuscript and confirmed that the average size of Gag puncta in each M-Sec-293 cell was larger than that in each Cr-293 cell (left panel), which was associated with the decrease of the total number of Gag puncta in each M-Sec-293 cell (right panel). To explain the results and experimental procedures, we added the following sentences (underlined):

[Page 8, lines 13 - 18]

Moreover, when transfected with the Gag expression plasmid [35] in place of the molecular clone, M-Sec-293 cells formed larger Gag puncta than Cr cells (Fig 4A). The average size of Gag puncta in each M-Sec-293 cell was larger than that in each Cr-293 cell (Fig 4B, left), which was associated with the decrease of the total number of Gag puncta in each M-Sec-293 cell (Fig 4B, right). The expression level of Gag in M-Sec-293 cells was comparable to that in Cr-293 cells (Fig 4C).

[The legend of Fig 4]

(B) The cells were analyzed as in **A**. *Left panel*, the average size of Gag puncta in each Gag^{+} cell is summarized (10 cells for each group). *Right panel*, the number of Gag puncta in each Gag^{+} cell is summarized (10 cells for each group). The Gag signal larger than approximately 0.5 μm was considered puncta. $^{*}p < 0.05$.

2. Figure 6: representative immunofluorescence experiments are missing.

Reply

According to the suggestion, we showed the typical images in Fig 6C. When compared to cells that did not express 5ptaseIV (−), cells expressing 5ptaseIV (+) had smaller Gag puncta (Fig 6C). To explain the results and experimental procedures, we added the following words and sentences (underlined):

[Page 9, lines 19 - 21]

When M-Sec-293 cells were co-transfected with the Gag expression plasmid and the wild-type 5ptaseIV plasmid, the size of Gag puncta became smaller (Fig 6C, Fig 6D, left).

[Page 20, lines 15 - 17 (Materials and methods)]

The HA-tagged Gag expression plasmid pCRVI/HTLV-1/Gag-HA [35] was used for the co-staining of Gag and Myc-tagged 5ptaseIV (Fig 6C).

[Page 20, lines 25 - page 21, line 3 (Materials and methods)]

For the co-staining of Gag and 5ptaseIV (Fig 6C), cells were stained with anti-HA (C29F4; Cell Signaling Technology) (to detect HA-tagged Gag) and anti-Myc antibodies (9E10; Santa Cruz Biotechnology) (to detect Myc-tagged 5ptaseIV) followed by

AlexaFluor568-labeled anti-rabbit IgG and AlexaFluor488-labeled anti-mouse IgG (both from Molecular Probes). Cells were also stained with phalloidin conjugated to AlexaFluor405 (Molecular Probes).

[The legend of Fig 6]

(C) M-Sec-293 cells were co-transfected with the Gag expression plasmid pCRVI/HTLV-1/Gag-HA (0.5 μg) and the WT 5ptaseIV plasmid (0.5 μg), cultured for 2 days, and analyzed for Gag (red), 5ptaseIV (green) or F-actin (blue) by immunofluorescence. The 5ptaseIV-positive (+) and negative (−) cells are indicated by arrows.

3. *Does M-Sec also lead to Gag aggregates during HIV-1 infection? If there is a difference/or not between the two viruses, it would be interesting to discuss.*

Reply

We thank for the comment. We are now carefully analyzing how M-Sec and its downstream effector molecules affect the aggregate formation of HIV-1 Gag and therefore added the following sentence (underlined):

[Page 14, lines 9 - 16]

The previous studies have suggested that HTLV-1 Gag is less dependent on PIP2 for its puncta formation than HIV-1 Gag [18, 19]. This difference may be because HTLV-1 Gag, but not HIV-1 Gag, binds both PIP2 and other acidic lipids such as phosphatidylserine [18, 19]. Meanwhile, M-Sec promotes the intracellular accumulation of PIP2. Under such conditions, HTLV-1 Gag can be attracted to the PIP2-rich compartments because the compartments are highly negatively charged. How M-Sec promotes the accumulation of HIV-1 Gag puncta to the PIP2-rich compartments is under investigation.

Reviewer #2

Part I - Summary

Masateru Hiyoshi and colleagues from Shinya Suzu's team previously reported (PLoS Pathog 2019) that HTLV-I Tax up-regulates M-Sec, a cellular protein involved in the formation of tunneling nanotubes and increasing cell motility. In the absence of M-Sec, the formation of clusters of HTLV-I Gag is reduced and viral cell-to-cell transmission is impaired. In this follow-up study, the authors study the underlying mechanisms. They now report that Gag cluster accumulation by M-Sec is dependent on phosphatidylinositol 4,5-bisphosphate (PIP2), since it is lost upon removal of PIP2 binding motifs in M-Sec or the depletion of cellular PIP2. They also show that Env co-localizes with the large Gag puncta, and that viral particles produced in the presence of M-Sec contains higher levels of Env.

Experiments are well performed. The authors use a variety of experimental systems, including Jurkat cells, 293T cells and primary T cells. This study is of interest, providing a mechanistic view of how HTLV-I highjacks M-Sec to facilitate viral cell-to-cell spread.

Part II - Major Issues

1. *The role of M-Sec on Env localization and virus incorporation is studied in 293T cells stably overexpressing M-Sec. Is M-Sec already present at basal levels in control 293T cells? It would be of importance to confirm these results in a more relevant system, such as a chronically infected T cell line. Is it possible to silence M-Sec in such cells and study the consequences on Env?*

Reply

M-Sec is not detectable in 293 cells, in our western blotting. According to the suggestions, we performed additional experiments using HTLV-1-infected M-Sec^{+} T cell lines (SLB-1 and MT-2), and confirmed that the M-Sec knockdown in these cells reduced the Env/Gag co-localization and the amount of Env in virus-like particles, as shown in the new figures (Fig 10 and S7 Fig). To explain the results and experimental procedures, we added the following sentences (underlined):

[Page 12, lines 3 - 9]

<u>Finally, we performed experiments using HTLV-1-infected M-Sec^{+} T cell lines, SLB-1 and MT-2. We previously reported that the knockdown of M-Sec in these cells altered the intracellular localization of Gag [16]. In this study, we found that the M-Sec knockdown in these cells did not affect the cell surface expression of the HTLV-1 receptor Glut1 and Env (S6 Fig), but reduced the Env/Gag co-localization (Fig 10A, Fig 10B, S7 Fig) and the amount of Env in VLPs (Fig 10C). These results further suggest that M-Sec promotes</u>

the incorporation of Env into viral particles.

[Page 19, lines 11 - 16 (Materials and methods)]

SLB-1 and MT-2 cells

SLB-1 cells were provided by M. Fujii (Niigata University, Japan). MT-2 cells were obtained from the Japanese Collection of Research Bioresources Cell Bank. These cells were maintained with RPMI1640 medium/10% FCS. The knockdown of M-Sec in these cells were performed using MISSION shRNA lentiviral transduction particles (Sigma), as described previously [16].

[The legend of Fig 10]

Fig 10. Effect of M-Sec knockdown on Env/Gag co-localization and Env incorporation into virus-like particles in SLB-1 and MT-2 cells.

(A) The control (Cr) SLB-1 cells or M-Sec knockdown (ΔM-Sec) SLB-1 cells were analyzed for Gag (green), Env (red) or F-actin (blue) by immunofluorescence. In right panels, the magnified images of "a", "b", "c" and "d" in the left and middle panels are shown. Scale bar: 10 μm (left and middle panels) or 5 μm (right panels).

(B) Pearson's correlation coefficients between Gag and Env are shown. In the left panel, the control (Cr) SLB-1 cells and M-Sec knockdown (ΔM-Sec) SLB-1 cells were analyzed. In the right panel, the control (Cr) MT-2 cells and M-Sec knockdown (ΔM-Sec) MT-2 cells were analyzed (see S7 Fig for immunofluorescence). $^{*}p < 0.05$.

(C) The virus-like particles (VLPs) in the supernatants were collected and analyzed for the amount of Env by western blotting after normalization of the Gag amount. In left blots, the control (Cr) SLB-1 cells and M-Sec knockdown (ΔM-Sec) SLB-1 cells were analyzed. In right blots, the control (Cr) MT-2 cells and M-Sec knockdown (ΔM-Sec) MT-2 cells were analyzed.

[The legend of S7 Fig]

S7 Fig. Effect of M-Sec knockdown on Env/Gag co-localization in MT-2 cells.

The control (Cr) MT-2 cells or M-Sec knockdown (ΔM-Sec) MT-2 cells were analyzed for Gag (green), Env (red) or F-actin (blue) by immunofluorescence. In right panels, the magnified images of "a", "b", "c" and "d" in the left and middle panels are shown. Scale bar: 10 μm (left and middle panels) or 5 μm (right panels).

2. Size and number of Gag puncta. In Fig. 3c, there are only five dots per condition. Does this mean that only 5 cells were analyzed? This needs to be clarified.

Reply

In the original Fig 3C, we analyzed 5 cells for each group. In the experiment, cells were transfected with the HTLV-1 molecular clone and therefore expressed Env. Because

HTLV-1 Env is highly fusogenic [references 32-34], the transfected cells in the experiment were fused and giant, and contained many Gag puncta in each giant cell (Fig 3B, middle panels). According to the comment, we analyzed total 10 cells for each group (the revised Fig 3C), and confirmed that the average size of Gag puncta in each giant M-Sec-293 cell was larger than that in each giant Cr-293 cell (left panel), which was associated with the decrease of the total number of Gag puncta in each giant M-Sec-293 cell (right panel). To clarify the point, we modified the legend of Fig 3 as follows (underlined):

[The legend of Fig 3]

(C) The cells were analyzed as in B. *Left panel*, the average size of Gag puncta in each Gag^+ giant cell is summarized (10 cells for each group). *Right panel*, the number of Gag puncta in each Gag^+ giant cell is summarized (10 cells for each group). The Gag signal larger than approximately 0.5 μm was considered puncta. $^*p < 0.05$.

3. In 293 T cells overexpressing M-Sec, what are the consequences on cellular shape and TNT formation?

Reply

According to the suggestion, we showed the results in the new figure (S3 Fig). M-Sec-expressing 293 cells formed longer membrane protrusions and showed smaller cell surface area than the control 293 cells, which was consistent with our previous findings that the knockdown of M-Sec in the T cell line MT-2 reduced the membrane protrusions and increased the cell surface area [reference 16]. To explain the results and experimental procedures, we added the following sentences or words (underlined):

[Page 8, lines 1 - 6]

To confirm that M-Sec promotes the accumulation of large Gag puncta, we established 293 cells stably expressing M-Sec (Fig 3A). M-Sec-293 cells formed longer membrane protrusions and showed smaller cell surface area than the control (Cr) 293 cells (S3 Fig), which was consistent with the previous findings that the knockdown of M-Sec in the T cell line MT-2 reduced the membrane protrusions and increased the cell surface area [16].

[Page 21, lines 5 - 7 (Materials and methods)]

The size of Gag puncta or membrane protrusions, or cell surface area was measured using ImageJ 1.52n software.

[The legend of S3 Fig]

S3 Fig. Size of membrane protrusions and cell surface area of M-Sec-expressing 293 cells. The control (Cr) 293 cells or 293 cells stably expressing M-Sec were stained with phalloidin (to visualize F-actin) and analyzed for the average size of membrane

protrusions in each cell (upper panel, 25 cells for each group) or the cell surface area (lower panel, 30 cells for each group). $^{*}p < 0.05$.

4. Does M-Sec affect surface levels of Env or of the HTLV-I receptor?

Reply

According to the suggestion, we performed an additional experiment using the control and M-Sec knockdown SLB-1 cells, and found that the M-Sec knockdown did not affect the cell surface expression of the HTLV-1 receptor Glut1 and Env in the cells, as shown in the new figure (S6 Fig). To explain the results and experimental procedures, we added the following sentences (underlined):

[Page 12, lines 3 - 9]

Finally, we performed experiments using HTLV-1-infected M-Sec^{+} T cell lines, SLB-1 and MT-2. We previously reported that the knockdown of M-Sec in these cells altered the intracellular localization of Gag [16]. In this study, we found that the M-Sec knockdown in these cells did not affect the cell surface expression of the HTLV-1 receptor Glut1 and Env (S6 Fig), but reduced the Env/Gag co-localization (Fig 10A, Fig 10B, S7 Fig) and the amount of Env in VLPs (Fig 10C). These results further suggest that M-Sec promotes the incorporation of Env into viral particles.

[Page 21, line 27 - page 22, line 3 (Materials and methods)]

SLB-1 cells were analyzed for the cell surface expression of Glut1 or Env by flow cytometry, using APC-labeled anti-Glut1antibodies (202915; R&D Systems) or anti-Env antibodies (LAT-27) [50] followed by APC-labeled anti-rat IgG antibodies (BD Biosciences).

[The legend of S6 Fig]

S6 Fig. Effect of M-Sec knockdown on cell surface expression of Glut1 and Env in SLB-1 cells.

The control (Cr) SLB-1 cells or M-Sec knockdown (ΔM-Sec) SLB-1 cells were analyzed for the cell surface expression of Glut1 (upper panel) or Env (lower panel) by flow cytometry (n=3). The expression level shown is relative to that of Cr SLB-1 cells. *n.s.*, not significant.

Reviewer #3

Part I - Summary

A previous study showed that the cellular protein M-Sec promotes the transmission of human T-cell leukemia virus type 1 (HTLV-1) in vitro and in vivo. In the current study, the authors investigate how HTLV-1 utilizes M-Sec for its efficient transmission. First, the authors show that HTLV-1-infected CD4+ T cells expressed M-Sec at a higher level than uninfected CD4+ T cells. They report that viral structural protein Gag is distributed in a punctate pattern in cells, where the M-Sec promoted the accumulation of large intracellular Gag puncta. The accumulation of large Gag puncta was dependent on PIP2; this was lost upon the removal of PIP2 binding motifs in M-Sec or the depletion of cellular PIP2. Env was co-localized with the large Gag puncta induced by M-Sec. Virus particles produced by M-Sec-expressing cells contained a higher amount of Env. The authors suggest that since M-Sec alters the cellular distribution of PIP2, M-Sec likely promotes the formation of infectious viral particles through PIP2. As M-Sec expression is mediated by HTLV-1 Tax protein, the authors conclude that M-Sec functions in a positive feedback loop to help ensure efficient HTLV-1 transmission.

There is novelty to the study, but there are numerous experimental issues that need to be addressed.

Part II - Major Issues

1. *In Figures 1 and 2, the authors show changes in mRNA levels, but not protein levels. The authors need to show that the protein levels are being changed as they are inferring based upon the RNA level changes to provide a convincing argument.*

Reply

According to the suggestion, we performed an additional experiment and confirmed the upregulation of M-Sec at the protein level, as shown in the new figure (Fig 2E). To explain the result and experimental procedures, we added the following sentences (underlined):

[Page 7, lines 10 - 17]

When their data deposited in the public NCBI Gene Expression Omnibus (#GSE234450) were analyzed, 4-1BB and HIAP were upregulated as early as 1 day after the ex vivo culture but declined thereafter (Fig 2D), the kinetics of which was similar to that of the expression of Tax mRNA [28]. In contrast, M-Sec mRNA was slowly but steadily upregulated at least up to day 6 (Fig 2D). <u>Consistent with this, M-Sec protein was steadily upregulated at least up to day 6 (Fig 2E).</u> These results suggest that HTLV-1 infection causes a sustained expression of M-Sec in $CD4^+$ T cells.

[The legend of Fig 2]

(E) Monocytes present in PBMCs of individuals with HAM/TSP (#4949 and #5061) were depleted by the adherence to culture dishes for 30 min. The monocytes-depleted PBMCs were cultured with RPMI1640 medium/10% FCS containing rhIL-2 for 1, 3 or 6 days, and analyzed for the expression of M-Sec protein by western blotting. β-actin blot is the loading control.

2-1. The large intracellular HTLV-1 Gag shown in Figures 3 and 4 in the presence of M-Sec expression is likely not contributing to virus particle production as it is not at the plasma membrane and is more likely an aggregate that is not biologically relevant to productive particle production, let alone infectious particle projection.

Reply

As pointed out, the large Gag puncta formed in the presence of M-Sec do not localize at the plasma membrane (Fig 3, Fig 4). Given the co-localization with Env (Fig 8), the large Gag puncta likely represent Gag localization at intracellular membranes rather than non-membrane-bound Gag aggregates. The small Gag puncta observed in the absence of M-Sec also localize at intracellular membranes (Fig 3B, Fig 4A). Previous reports showed that such localization is driven by the HTLV-1 MA domain because HIV-1 Gag chimeric constructs in which its MA is replaced with HTLV-1 MA show extensive intracellular distribution in contrast to HIV-1 Gag, which mainly localizes at the plasma membrane [references 18 and 19]. Importantly, despite this difference in subcellular localization, HTLV-1 Gag and HIV-1 Gag chimeras containing the HTLV-1 MA domain release viral particles at even higher efficiency (i.e., released Gag versus total Gag) than HIV-1 Gag with its native MA [references 18 and 19]. These findings are consistent with the idea that the large HTLV-1 Gag puncta that localize at intracellular compartments contribute to the formation of infectious viral particles, although more detailed analyses are required. To discuss these points, we added the following sentences (underlined):

[Page 15, line 18 - page 16, line 8]

Thus, it is reasonable to speculate that the Gag puncta recruit Env through direct or indirect interaction, which facilitates the Gag/Env co-localization. This idea is consistent with the finding that the amount of Env in VLPs produced by M-Sec-expressing cells is higher than that by the control cells (Fig 9). Such Gag/Env co-localization may serve as platforms for the formation of virus particles and facilitate the transmission of these particles since it was reported that Gag and Env accumulate at the site of tight cell-to-cell contact and that Gag and Env are rapidly transferred to the target cell [1]. The large Gag puncta formed in the presence of M-Sec localized at intracellular compartments, but not at the plasma membrane (Fig 3, Fig 4). The small Gag puncta in the absence of M-Sec

also localized at intracellular compartments (Fig 3, Fig 4), as reported previously [18]. Such localization is unique to HTLV-1 Gag because HIV-1 Gag mainly localized at the plasma membrane [18]. Interestingly, despite this difference in subcellular localization, which is determined by the MA domains [18, 19], not only HIV-1 Gag but also HTLV-1 Gag and HIV-1 Gag chimera containing HTLV-1 MA efficiently release viral particles [18, 19]. These findings are consistent with the idea that the large HTLV-1 Gag puncta that localize at intracellular compartments contribute to the formation of infectious viral particles, although more detailed analyses of HTLV-1 Gag trafficking are required.

2-2. In Figure 4B, the changes in Gag expression needs to be quantified. It appears that the difference is likely not significant.

Reply

As pointed out, the expression level of Gag in M-Sec-293 cells was comparable to that in Cr-293 cells (Fig 4C in the revised manuscript), which was further confirmed by the quantitative analysis using independent experiments, as shown below.

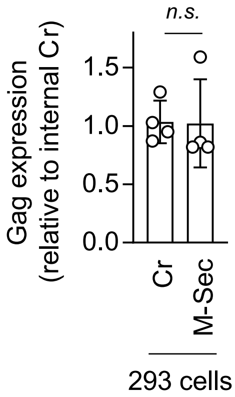

Because M-Sec-293 cells formed larger Gag puncta than Cr-293cells (Fig 4A), these results suggest that M-Sec promotes the accumulation of the large Gag puncta without affecting the expression level of Gag. To clarify the point, we added the following words (underlined):

[Page 8, lines 13 - 20]

Moreover, when transfected with the Gag expression plasmid [35] in place of the molecular clone, M-Sec-293 cells formed larger Gag puncta than Cr-293cells (Fig 4A). The average size of Gag puncta in each M-Sec-293 cell was larger than that in each Cr-293 cell (Fig 4B, left), which was associated with the decrease of the total number of Gag puncta in each M-Sec-293 cell (Fig 4B, right). The expression level of Gag in M-Sec-293 cells was comparable to that in Cr-293 cells (Fig 4C). These results suggest that M-Sec promotes the accumulation of the large Gag puncta without affecting the expression level

of Gag, and that other viral components are dispensable for the function of M-Sec.

3. *Figure 5 shows that the M-Sec deletion mutants change the trends in large punctate Gag. How this results in any change(s) in virus particle production or particle infectivity is not shown. This figure has limited interpretative power in terms of biological relevance.*
4. *Figure 6 has the same issue as not showing the connection between Gag puncta size and its biological relevance to virus particle production and virus infectivity.*

Reply to comments-3 and 4

According to the suggestions, we performed additional experiments and confirmed the connection between Gag puncta size and Env incorporation into viral particles, as shown in the new figure (Fig 9D). Specifically, we found that the amount of Env in virus-like particles (VLPs) produced by 293 cells expressing the M-Sec mutant (ΔK1 or ΔK2) was lower than that produced by 293 cells expressing the wild-type M-Sec (Fig 9D, upper half). We also found that the amount of Env in VLPs produced by M-Sec-293 cells transfected with the wild-type 5ptaseIV plasmid was lower than that produced by M-Sec-293 cells transfected with the empty vector or the Δ1 5ptaseIV mutant (Fig 9D, lower half). To explain these results and experimental procedures, we added the following sentences (underlined):

[Page 11, lines 20 - 26]

The amount of Env in VLPs produced by 293 cells expressing the M-Sec mutants (ΔK1 or ΔK2), both of which failed to promote the accumulation of the large Gag puncta (Fig 5), was lower than that produced by 293 cells expressing the wild-type M-Sec (Fig 9D, upper half). The amount of Env in VLPs produced by M-Sec-293 cells transfected with the wild-type 5ptaseIV plasmid, which inhibited the M-Sec-mediated accumulation of large Gag puncta (Fig 6), was also lower than that produced by M-Sec-293 cells transfected with the empty vector or the Δ1 5ptaseIV mutant (Fig 9D, lower half).

[The legend of Fig 9]

(D) *Upper half*, 293 cells expressing the wild-type (WT) or mutant M-Sec (ΔK1 and ΔK2) were co-transfected with the Gag expression plasmid (0.3 μg), the Env expression plasmid (0.3 μg) and the Rev expression plasmid (0.4 μg), and cultured for 2 days. The VLPs in the supernatants were collected and analyzed for the amount of Env or Gag by western blotting. *Lower half*, 293 cells expressing the wild-type M-Sec were co-transfected with the Gag expression plasmid (0.3 μg), the Env expression plasmid (0.3 μg), the Rev expression plasmid (0.3 μg), and one of the following plasmids; the empty vector (0.1 μg), the wild type 5ptaseIV plasmid (0.1 μg) or the Δ1 mutant 5ptaseIV

plasmid (0.1 μg). The cells were cultured for 1 day, and the VLPs in the supernatants were collected and analyzed for the amount of Env or Gag by western blotting.

5. *Figures 8 and 9 are missing evidence that the level of M-Sec expression in the 293 cells are physiologically relevant to that see in CD4+ T cells. What would be helpful are control experiments in which the M-Sec plasmid is titrated into cells.*

Reply

As pointed out, it is important to confirm our conclusions using cells expressing M-Sec at physiological levels. In the revised manuscript, we showed the results of HTLV-1-infected T cell lines (SLB-1 and MT-2) because we previously established the M-Sec knockdown SLB-1 or MT-2 cells [reference 16]. As shown in the new figures (Fig 10, S7 Fig), we confirmed that the M-Sec knockdown in these cells reduced the Env/Gag co-localization and the amount of Env in virus-like particles, as shown in the new figures (Fig 10 and S7 Fig). To explain the results and experimental procedures, we added the following sentences (underlined):

[Page 12, lines 3 - 9]

Finally, we performed experiments using HTLV-1-infected M-Sec$^+$ T cell lines, SLB-1 and MT-2. We previously reported that the knockdown of M-Sec in these cells altered the intracellular localization of Gag [16]. In this study, we found that the M-Sec knockdown in these cells did not affect the cell surface expression of the HTLV-1 receptor Glut1 and Env (S6 Fig), but reduced the Env/Gag co-localization (Fig 10A, Fig 10B, S7 Fig) and the amount of Env in VLPs (Fig 10C). These results further suggest that M-Sec promotes the incorporation of Env into viral particles.

[Page 19, lines 11 - 16 (Materials and methods)]

SLB-1 and MT-2 cells

SLB-1 cells were provided by M. Fujii (Niigata University, Japan). MT-2 cells were obtained from the Japanese Collection of Research Bioresources Cell Bank. These cells were maintained with RPMI1640 medium/10% FCS. The knockdown of M-Sec in these cells were performed using MISSION shRNA lentiviral transduction particles (Sigma), as described previously [16].

[The legend of Fig 10]

Fig 10. Effect of M-Sec knockdown on Env/Gag co-localization and Env incorporation into virus-like particles in SLB-1 and MT-2 cells.

(A) The control (Cr) SLB-1 cells or M-Sec knockdown (ΔM-Sec) SLB-1 cells were analyzed for Gag (green), Env (red) or F-actin (blue) by immunofluorescence. In right panels, the magnified images of "a", "b", "c" and "d" in the left and middle panels are

shown. Scale bar: 10 μm (left and middle panels) or 5 μm (right panels).
(B) Pearson's correlation coefficients between Gag and Env are shown. In the left panel, the control (Cr) SLB-1 cells and M-Sec knockdown (ΔM-Sec) SLB-1 cells were analyzed. In the right panel, the control (Cr) MT-2 cells and M-Sec knockdown (ΔM-Sec) MT-2 cells were analyzed (see S7 Fig for immunofluorescence). $^{*}p < 0.05$.
(C) The virus-like particles (VLPs) in the supernatants were collected and analyzed for the amount of Env by western blotting after normalization of the Gag amount. In left blots, the control (Cr) SLB-1 cells and M-Sec knockdown (ΔM-Sec) SLB-1 cells were analyzed. In right blots, the control (Cr) MT-2 cells and M-Sec knockdown (ΔM-Sec) MT-2 cells were analyzed.
[The legend of S7 Fig]
S7 Fig. Effect of M-Sec knockdown on Env/Gag co-localization in MT-2 cells.
The control (Cr) MT-2 cells or M-Sec knockdown (ΔM-Sec) MT-2 cells were analyzed for Gag (green), Env (red) or F-actin (blue) by immunofluorescence. In right panels, the magnified images of "a", "b", "c" and "d" in the left and middle panels are shown. Scale bar: 10 μm (left and middle panels) or 5 μm (right panels).

Part III - Minor Issues

1. Image resolution needs to be improved.

Reply

We hope that the resolution of images has been improved because we checked our figures using the PLOS-provided tool PACE before the revision.
