# Supplementary material for: M-Sec promotes the accumulation of intracellular HTLV-1 Gag puncta and the incorporation of Env into viral particles
Source: PLoS Pathog. 2025 Jan 27;21(1):e1012919. doi: 10.1371/journal.ppat.1012919 (PMC11801699; doi:10.1371/journal.ppat.1012919)
Supplement: Peer review history

# Peer review: Decision Letter 0

**DOI:** 10.1371/journal.ppat.1012919.r001

**Authors:** Charles R M Bangham, Susan R. Ross

26 Aug 2024

Dear Dr Suzu,

Thank you very much for submitting your manuscript "M-Sec promotes the accumulation of intracellular HTLV-1 Gag puncta and the incorporation of Env into viral particles" for consideration at PLOS Pathogens. As with all papers reviewed by the journal, your manuscript was reviewed by members of the editorial board and by several independent reviewers. In light of the reviews (below this email), we would like to invite the resubmission of a significantly-revised version that takes into account the reviewers' comments.

All three reviewers found your results of both interest and potential importance in understanding the production and transmission of HTLV-1 virions. However, all three also had specific reservations and requirements concerning certain aspects of the work. In particular, the reviewers shared the view that it was necessary to include experiments on HTLV-1-infected T lymphocytes, and not to rely exclusively on 293T cells; and several suggestions and requirements were made concerning the design and interpretation of the microscopy experiments.

We cannot make any decision about publication until we have seen the revised manuscript and your response to the reviewers' comments. Your revised manuscript is also likely to be sent to reviewers for further evaluation.

When you are ready to resubmit, please upload the following:

[1] A letter containing a detailed list of your responses to the review comments and a description of the changes you have made in the manuscript. Please note while forming your response, if your article is accepted, you may have the opportunity to make the peer review history publicly available. The record will include editor decision letters (with reviews) and your responses to reviewer comments. If eligible, we will contact you to opt in or out.

[2] Two versions of the revised manuscript: one with either highlights or tracked changes denoting where the text has been changed; the other a clean version (uploaded as the manuscript file).

Important additional instructions are given below your reviewer comments.

Please prepare and submit your revised manuscript within 60 days. If you anticipate any delay, please let us know the expected resubmission date by replying to this email. Please note that revised manuscripts received after the 60-day due date may require evaluation and peer review similar to newly submitted manuscripts.

Thank you again for your submission. We hope that our editorial process has been constructive so far, and we welcome your feedback at any time. Please don't hesitate to contact us if you have any questions or comments.

Sincerely,

Charles

Charles R M Bangham, ScD FRS

Academic Editor

PLOS Pathogens

Susan Ross

Section Editor

PLOS Pathogens

Michael Malim

Editor-in-Chief

PLOS Pathogens

orcid.org/0000-0002-7699-2064

***********************

All three reviewers found your results of both interest and potential importance in understanding the production and transmission of HTLV-1 virions. However, all three also had specific reservations and requirements concerning certain aspects of the work. In particular, the reviewers shared the view that it was necessary to include experiments on HTLV-1-infected T lymphocytes, and not to rely exclusively on 293T cells; and several suggestions and requirements were made concerning the design and interpretation of the microscopy experiments.

Reviewer's Responses to Questions

**Part I - Summary**

Please use this section to discuss strengths/weaknesses of study, novelty/significance, general execution and scholarship.

Reviewer #1: Hiyoshi et al., previously reported that M-Sec, a cellular protein involved in the formation of tunneling nanotubes, is overexpressed in HTLV-1 infected cells. They reported that the viral protein Tax activates the transcription of M-Sec, and that M-Sec promotes HTLV-1 infection through increase in membrane protrusions and cell motility. In this manuscript, “M-Sec promotes the accumulation of intracellular HTLV-1 Gag puncta and the incorporation of Env into viral particles”, the same authors further investigate how M-Sec could stimulate HTLV-1 infection. They show that M-Sec interacts with phosphatidylinositol 4,5-biphosphate (PIP2), through a lysine-rich motif, which promotes the formation of Gag puncta in the cells. They then found that these Gag aggregates lead to Env accumulation, which consequently increased incorporation of Env into viral particles. This is a very interesting study that increases our knowledge of cellular proteins important for HTLV-1 virion formation. Some additional experiments, listed below, are necessary to strengthen the conclusion of the manuscript.

Reviewer #2: Masateru Hiyoshi and colleagues from Shinya Suzu’s team previously reported (Plos Path 2019) that HTLV-I Tax up-regulates M-Sec, a cellular protein involved in the formation of tunneling nanotubes and increasing cell motility. In the absence of M-Sec, the formation of clusters of HTLV-I Gag is reduced and viral cell-to-cell transmission is imapired. In this follow-up study, the authors study the underlying mechanisms. They now report that Gag cluster accumulation by M-Sec is dependent on phosphatidylinositol 4,5-bisphosphate (PIP2), since it is lost upon removal of PIP2 binding motifs in M-Sec or the depletion of cellular PIP2. They also show that Env co-localizes with the large Gag puncta, and that viral particles produced in the presence of M-Sec contains higher levels of Env.

Experiments are well performed. The authors use a variety of experimental systems, inlucing Jurkat cells, 293T cells and primary T cells. This study is of interest, providing a mechanistic view of how HTLV-I highjacks M-Sec to facilitate viral cell-to-cell spread.

Reviewer #3: A previous study showed that the cellular protein M-Sec promotes the transmission of human T-cell leukemia virus type 1 (HTLV-1) in vitro and in vivo. In the current study, the authors investigate how HTLV-1 utilizes M-Sec for its efficient transmission. First, the authors show that HTLV-1-infected CD4+ T cells expressed M-Sec at a higher level than uninfected CD4+ T cells. They report that viral structural protein Gag is distributed in a punctate pattern in cells, where the M-Sec promotes promoted the accumulation of large intracellular Gag puncta. The accumulation of large Gag puncta was dependent on PIP2; this was lost upon the removal of PIP2 binding motifs in M-Sec or the depletion of cellular PIP2. Env was co-localized with the large Gag puncta induced by M-Sec. Virus particles produced by M-Sec-expressing cells contained a higher amount of Env. The authors suggest that since M-Sec alters the cellular distribution of PIP2, M-Sec likely promotes the formation of infectious viral particles through PIP2. As M-Sec expression is mediated by HTLV-1 Tax protein, the authors conclude that M-Sec functions in a positive feedback loop to help ensure efficient HTLV-1 transmission.

There is novelty to the study, but there are numerous experimental issues that need to be addressed.

**********

**Part II – Major Issues: Key Experiments Required for Acceptance**

Please use this section to detail the key new experiments or modifications of existing experiments that should be absolutely required to validate study conclusions.

Generally, there should be no more than 3 such required experiments or major modifications for a "Major Revision" recommendation. If more than 3 experiments are necessary to validate the study conclusions, then you are encouraged to recommend "Reject".

Reviewer #1: 1. One major concern is that the authors exclusively use 293 cells to analyze the co-localization of PIP2/Gag/Env in presence or absence of M-Sec. The conclusion of the manuscript will be stronger/more pertinent if some microscopy experiments are also performed in HTLV-1 infected T-cells before and after knock-down of M-Sec.

2. Same concern for figure 9, knock-down of M-Sec should be performed in HTLV-1 infected cells to evaluate its effect on Env incorporation into virions.

3. Another concern is that M-Sec localization in HTLV-1 infected T- cells is never shown. It will be interesting to determine its localization and whether it colocalizes with Gag/Env in these cells. The authors used an M-Sec antibody for immunofluorescence (F-6; Santa Cruz Biotechnology) in a previous publication regarding the role of M-Sec in HIV-1 infection.

4. Figure 2D should also show Tax mRNA expression (available in the data set).

Reviewer #2: Main concerns

1. The role of Msec on Env localization and virus incorporation is studied in 293T cells stably overexpressing M-Sec. Is M-Sec already present at basal levels in control 293T cells? It would be of importance to confirm these results in a more relevant system, such as a chronically infected T cell line. Is it possible too silence M-sec in such cells and study the consequences on Env ?

2. Size and number of Gag puncta. In Fig. 3b, there are only five dots per condition. Does this mean that only 5 cells were analyzed? This needs to be clarified.

3. In 293 T cells overexpressing M-Sec, what are the consequences on cellular shape and TNT formation?

4. Does M-Sec affect surface levels of Env or of the HTLV-I receptor?

Reviewer #3: 1. In Figures 1 and 2, the authors show changes in mRNA levels, but not protein levels. The authors need to show that the protein levels are being changed as they are inferring based upon the RNA level changes to provide a convincing argument.

2. The large intracellular HTLV-1 Gag shown in Figures 3 and 4 in the presence of M-Sec expression is likely not contributing to virus particle production as it is not at the plasma membrane and is more likely an aggregate that is not biologically relevant to productive particle production, let alone infectious particle projection. In Figure 4B, the changes in Gag expression needs to be quantified. It appears that the difference is likely not significant.

3. Figure 5 shows that the M-Sec deletion mutants change the trends in large punctate Gag. How this results in any change(s) in virus particle production or particle infectivity is not shown. This figure has limited interpretative power in terms of biological relevance.

4. Figure 6 has the same issue as not showing the connection between Gag puncta size and its biological relevance to virus particle production and virus infectivity.

5. Figures 8 and 9 are missing evidence that the level of M-Sec expression in the 293 cells are physiologically relevant to that see in CD4+ T cells. What would be helpful are control experiments in which the M-Sec plasmid is titrated into cells.

**********

**Part III – Minor Issues: Editorial and Data Presentation Modifications**

Please use this section for editorial suggestions as well as relatively minor modifications of existing data that would enhance clarity.

Reviewer #1: 1. Figure 4: quantification is missing

2. Figure 6: representative immunofluorescence experiments are missing.

3. Does M-Sec also lead to Gag aggregates during HIV-1 infection? If there is a difference/or not between the two viruses, it would be interesting to discuss.

Reviewer #2: (No Response)

Reviewer #3: Image resolution needs to be improved

**********

PLOS authors have the option to publish the peer review history of their article (what does this mean?). If published, this will include your full peer review and any attached files.

If you choose “no”, your identity will remain anonymous but your review may still be made public.

**Do you want your identity to be public for this peer review?** For information about this choice, including consent withdrawal, please see our Privacy Policy.

Reviewer #1: No

Reviewer #2: No

Reviewer #3: No

Figure Files:

While revising your submission, please upload your figure files to the Preflight Analysis and Conversion Engine (PACE) digital diagnostic tool, https://pacev2.apexcovantage.com. PACE helps ensure that figures meet PLOS requirements. To use PACE, you must first register as a user. Then, login and navigate to the UPLOAD tab, where you will find detailed instructions on how to use the tool. If you encounter any issues or have any questions when using PACE, please email us at figures@plos.org.

Data Requirements:

Please note that, as a condition of publication, PLOS' data policy requires that you make available all data used to draw the conclusions outlined in your manuscript. Data must be deposited in an appropriate repository, included within the body of the manuscript, or uploaded as supporting information. This includes all numerical values that were used to generate graphs, histograms etc.. For an example see here on PLOS Biology: http://www.plosbiology.org/article/info%3Adoi%2F10.1371%2Fjournal.pbio.1001908#s5.

Reproducibility:

To enhance the reproducibility of your results, we recommend that you deposit your laboratory protocols in protocols.io, where a protocol can be assigned its own identifier (DOI) such that it can be cited independently in the future. Additionally, PLOS ONE offers an option to publish peer-reviewed clinical study protocols. Read more information on sharing protocols at https://plos.org/protocols?utm_medium=editorial-email&utm_source=authorletters&utm_campaign=protocols

# Author comment: Author response to Decision Letter 0

**DOI:** 10.1371/journal.ppat.1012919.r002

24 Oct 2024

Attachment Submitted filename: Response to Reviewers (R1).pdf

# Peer review: Decision Letter 1

**DOI:** 10.1371/journal.ppat.1012919.r003

**Authors:** Charles R M Bangham, Susan R. Ross

17 Jan 2025

Dear Dr Suzu,

We are pleased to inform you that your manuscript 'M-Sec promotes the accumulation of intracellular HTLV-1 Gag puncta and the incorporation of Env into viral particles' has been provisionally accepted for publication in PLOS Pathogens.

Before your manuscript can be formally accepted you will need to complete some formatting changes, which you will receive in a follow up email. A member of our team will be in touch with a set of requests.

Please note that your manuscript will not be scheduled for publication until you have made the required changes, so a swift response is appreciated.

IMPORTANT: The editorial review process is now complete. PLOS will only permit corrections to spelling, formatting or significant scientific errors from this point onwards. Requests for major changes, or any which affect the scientific understanding of your work, will cause delays to the publication date of your manuscript.

Should you, your institution's press office or the journal office choose to press release your paper, you will automatically be opted out of early publication. We ask that you notify us now if you or your institution is planning to press release the article. All press must be co-ordinated with PLOS.

Thank you again for supporting Open Access publishing; we are looking forward to publishing your work in PLOS Pathogens.

Best regards,

Charles R M Bangham, ScD FRS

Academic Editor

PLOS Pathogens

Susan Ross

Section Editor

PLOS Pathogens

Michael Malim

Editor-in-Chief

PLOS Pathogens

orcid.org/0000-0002-7699-2064

***********************************************************

Reviewer Comments (if any, and for reference):

Reviewer's Responses to Questions

**Part I - Summary**

Please use this section to discuss strengths/weaknesses of study, novelty/significance, general execution and scholarship.

Reviewer #1: The authors responded adequately to the original comments. No further comments from this reviewer.

Reviewer #3: The authors have adequately addressed the reviewers comments and the manuscript is improved.

**********

**Part II – Major Issues: Key Experiments Required for Acceptance**

Please use this section to detail the key new experiments or modifications of existing experiments that should be absolutely required to validate study conclusions.

Generally, there should be no more than 3 such required experiments or major modifications for a "Major Revision" recommendation. If more than 3 experiments are necessary to validate the study conclusions, then you are encouraged to recommend "Reject".

Reviewer #1: None.

Reviewer #3: (No Response)

**********

**Part III – Minor Issues: Editorial and Data Presentation Modifications**

Please use this section for editorial suggestions as well as relatively minor modifications of existing data that would enhance clarity.

Reviewer #1: None.

Reviewer #3: (No Response)

**********

PLOS authors have the option to publish the peer review history of their article (what does this mean?). If published, this will include your full peer review and any attached files.

If you choose “no”, your identity will remain anonymous but your review may still be made public.

**Do you want your identity to be public for this peer review?** For information about this choice, including consent withdrawal, please see our Privacy Policy.

Reviewer #1: No

Reviewer #3: No

# Editor report: Acceptance letter

**DOI:** 10.1371/journal.ppat.1012919.r004

**Authors:** Charles R M Bangham, Susan R. Ross

23 Jan 2025

Dear Dr Suzu,

We are delighted to inform you that your manuscript, "M-Sec promotes the accumulation of intracellular HTLV-1 Gag puncta and the incorporation of Env into viral particles," has been formally accepted for publication in PLOS Pathogens.

We have now passed your article onto the PLOS Production Department who will complete the rest of the pre-publication process. All authors will receive a confirmation email upon publication.

The corresponding author will soon be receiving a typeset proof for review, to ensure errors have not been introduced during production. Please review the PDF proof of your manuscript carefully, as this is the last chance to correct any scientific or type-setting errors. Please note that major changes, or those which affect the scientific understanding of the work, will likely cause delays to the publication date of your manuscript. Note: Proofs for Front Matter articles (Pearls, Reviews, Opinions, etc...) are generated on a different schedule and may not be made available as quickly.

Soon after your final files are uploaded, the early version of your manuscript, if you opted to have an early version of your article, will be published online. The date of the early version will be your article's publication date. The final article will be published to the same URL, and all versions of the paper will be accessible to readers.

Thank you again for supporting open-access publishing; we are looking forward to publishing your work in PLOS Pathogens. 

Best regards,

Sumita Bhaduri-McIntosh

Editor-in-Chief

PLOS Pathogens

orcid.org/0000-0003-2946-9497

Michael Malim

Editor-in-Chief

PLOS Pathogens

orcid.org/0000-0002-7699-2064
